# Supplementary material for: Acceptability of aspirin for cancer preventive therapy: a survey and qualitative study exploring the views of the UK general population
Source: BMJ Open. 2023 Dec 18;13(12):e078703. doi: 10.1136/bmjopen-2023-078703 (PMC10748871; doi:10.1136/bmjopen-2023-078703)
Supplement: Supplementary data [file bmjopen-2023-078703supp001.pdf]

Supplementary Materials 1: Short survey on aspirin use for cancer prevention among the general public

1. Do you ever take aspirin?
   a. Yes
   b. No

2. Do you take aspirin regularly (i.e. most days or every day)?
   a. Yes
   b. No
   c. N/A – do not take aspirin

3. What is the main reason you take aspirin?
   a. Pain relief
   b. Prevention of cardiovascular disease (i.e. heart and circulatory disease)
   c. Prevention of cancer
   d. Other reason, please specify ______________
   e. N/A – do not take aspirin

4. Prior to completing this survey, were you aware that aspirin could reduce the risk of developing certain types of cancers?
   a. Yes
   b. No

5. Has your doctor or any other healthcare professional talked to you about how aspirin may lower your risk of developing bowel cancer or any other cancers?
   a. Yes
   b. No

6. Have you previously been diagnosed with cancer?
   a. Yes, please specify which cancer(s): _______________
   b. No

7. How would you describe your gender?
   a. Male
   b. Female
   c. Non-binary
   d. Different identity
   e. Prefer not to say

8. What is your age?

   ____

9. How would you describe your ethnicity?
   a. White - English/ Welsh/ Scottish/ Northern Irish/ British
   b. White - Irish

c. White - Gypsy or Irish Traveller
d. Any other White background
e. Mixed - White and Black Caribbean
f. Mixed - White and Black African
g. Mixed - White and Asian
h. Any other mixed/ multiple ethnic background
i. Asian or Asian British - Indian
j. Asian or Asian British - Pakistani
k. Asian or Asian British - Bangladeshi
l. Asian or Asian British - Chinese
m. Any other Asian background
n. Black or Black British - African
o. Black or Black British - Caribbean
p. Any other Black/ African/ Caribbean background
q. Arab or Arab British
r. Any other ethnic group

10. What is the highest level of educational or professional qualification you have obtained?
    a. GCSE/ O-level/ CSE
    b. Vocational qualifications (e.g. NVQ1+2)
    c. A-Level or equivalent (e.g. NVQ3)
    d. Bachelor’s degree or equivalent (e.g. NVQ4)
    e. Masters/ PhD or equivalent
    f. Other qualifications
    g. No formal qualifications

Thank you for taking part in the survey study. If you are interested in taking part further, we are looking for people to take part in a follow-up interview study to explore their thoughts on the use of aspirin for bowel cancer prevention. The interviews will take between 30 to 60 minutes to complete and will be take place either over the telephone or video call, depending upon your preference. You will receive £25 for taking part in the follow-up interview study. You do not need to use aspirin or know anything about this topic to take part.

11. Are you interested in taking part in the follow-up interview study?
    a. Yes, please provide your contact details (e.g. email, phone number) and we will contact you further about the study: ___________________
    b. No

Supplementary Materials 2: Interview schedule for general public interviews

Thank you for agreeing to take part. Just before we start the interview, I'd like to quickly go over some key points about your rights as a participant in this study. It is completely fine if at any point you would like to stop the interview, or if you do not want to answer a question, please just let me know. You can also withdraw from the study at any point and you do not have to provide a reason for why. Just to remind you the interview will be recorded, but anything you say to me today will be kept confidential, and if you are quoted in any of our reports your name will not be used and instead you will be given a pseudonym, which is a fake name. I will also anonymise anything identifiable, like places, you mention as well. After the interview, if you have any further questions about anything we discussed today, please feel free to contact me. I can also provide a debrief sheet at the end with website links with further information on this topic in case you are interested in reading more. Are you happy to continue?

General public interview questions

As you read on the information sheet, the study is aiming to understand what people think about using aspirin for cancer prevention, including the reasons why people may or may not be willing to use it. Aspirin has previously been found to effectively reduce people's risk of bowel cancer in a healthy population sample. At the moment, aspirin is only officially recommended by the NHS for cancer prevention in people who have a genetic condition that makes them higher risk of developing bowel cancer. There is potential in the future though for this medication to be offered more widely to the general public for the purpose of bowel cancer prevention.

While there is the potential benefit of having a reduced likelihood of developing bowel cancer in the future, there are also side-effects to regular aspirin use. The main side-effect is an increased likelihood of experiencing internal bleeding.

I will now move onto the interview questions, which will explore your thoughts on using aspirin regularly for cancer prevention. Just a reminder before we start, you do not need to know anything about the use of aspirin for cancer prevention to answer these questions. We are just interested in hearing your initial thoughts.

**Domains**	**Interview questions**
Knowledge<br><br>Enviromental context and resources<br><br>Intentions<br><br>Social influences	What are your initial thoughts on taking aspirin daily for bowel cancer prevention?<br><br>Had you heard of the use of aspirin for cancer prevention before this interview?<br><br>If yes, where and how did you hear about this?<br><br>If no, how would you find such information? Prompts: from what sources? Internet? A healthcare professional?<br><br>Have you ever been encouraged or discouraged by someone to take aspirin regularly for cancer prevention? Prompt: Why was this?
Optimism<br><br>Beliefs about consequences<br><br>Emotions<br><br>Enviromental context and resources<br><br>Skills<br><br>Beliefs about capabilities<br><br>Reinforcement	In your opinion, do you think using aspirin would be a good way to reduce your risk of developing bowel cancer in the future? Prompt: Why do you think this?<br><br>Do you have any concerns about taking aspirin regularly? Prompt: How do you feel about the side-effects?<br><br>What kind of information do you think you would need to help you make a decision on whether to take aspirin regularly for cancer prevention?<br><br>Where would you go to get this information? Prompts: What would your first step be to obtain this information? Internet? Healthcare professionals?<br><br>*[If they do take aspirin for cancer prevention]* Have you previously encountered any problems with trying to get further information on the use of aspirin for cancer prevention? If yes, please describe these problems.<br><br>*[If yes to above question]* Is there anything about this experience that makes you more or less likely to try and take aspirin regularly in the future?
Beliefs about consequences	I mentioned before one of potential side-effects of regular aspirin use can be internal bleeding. There are several risk factors that make a person more likely to experience internal bleeding from regular aspirin use. These include:<br><br>- Active or previous stomach ulcers<br>- Bleeding disorders

	- Being over the age of 65<br>- Uncontrolled hypertension<br>- Previous history of stroke<br>- Abnormal liver or renal function<br>- Experience indigestion on aspirin<br><br>If you had any of these risk factors, your doctor may offer you an additional medication to reduce your risk of internal bleeding.<br><br>What are your thoughts on taking regular aspirin alongside another medication to reduce your risk of bowel cancer?
Goals<br><br>Memory, attention and decision processes	How much of a priority is taking aspirin for cancer prevention to you? Prompts: how high or low a priority is it?<br><br>Are there any other higher priorities? Prompt: Prevention priorities? What are they?

Brief quantitative questions

Thank you so much for your answers so far. Just before we end the interview, there are several brief demographic questions that I would just like to go through with you. If you do not want to answer one of these questions, please just let me know and we can skip it.

1) What is your age?
2) Please describe your gender? Male; Female; Non-binary; Another identity; Prefer not to say.
3) How would you describe your ethnicity? Examples include White British, Indian, Mixed – White and Black Caribbean
4) What country in the UK do you live?
5) Have you previously been diagnosed with cancer?

Debrief

Thank you for all your help with answering my questions. We really appreciate your time and hope that it will be useful in the future when we are trying to support people when making a decision about whether to use aspirin for cancer prevention. Before we end, do you have any questions you would like to ask me?

If you would like more information on the use of aspirin for cancer prevention, I can email you an information sheet with links to website with further information on this topic. If you would like to ask me further questions on the study, please do not hesitate to contact me.
