# Supplementary material for: Acceptability of aspirin for cancer preventive therapy: a survey and qualitative study exploring the views of the UK general population
Source: BMJ Open. 2023 Dec 18;13(12):e078703. doi: 10.1136/bmjopen-2023-078703 (PMC10748871; doi:10.1136/bmjopen-2023-078703)
Supplement: Author's manuscript [file bmjopen-2023-078703.draft_revisions.pdf]

BMJ Open

BMJ Open is committed to open peer review. As part of this commitment we make the peer review history of every article we publish publicly available.

When an article is published we post the peer reviewers' comments and the authors' responses online. We also post the versions of the paper that were used during peer review. These are the versions that the peer review comments apply to.

The versions of the paper that follow are the versions that were submitted during the peer review process. They are not the versions of record or the final published versions. They should not be cited or distributed as the published version of this manuscript.

BMJ Open is an open access journal and the full, final, typeset and author-corrected version of record of the manuscript is available on our site with no access controls, subscription charges or pay-per-view fees (http://bmjopen.bmj.com).

If you have any questions on BMJ Open's open peer review process please email info.bmjopen@bmj.com

BMJ Open

Acceptability of aspirin for cancer preventive therapy: a mixed methods study exploring the views of the UK general population

Journal:	*BMJ Open*
Manuscript ID	bmjopen-2023-078703
Article Type:	Original research
Date Submitted by the Author:	09-Aug-2023
Complete List of Authors:	Lloyd, Kelly; University of Leeds Faculty of Medicine and Health, Leeds Institute of Health Sciences<br>Hall, Louise; University of Leeds Faculty of Medicine and Health, Leeds Institute of Health Sciences<br>Ziegler, Lucy; University of Leeds Faculty of Medicine and Health, Leeds Institute of Health Sciences<br>Foy, Robbie; University of Leeds Faculty of Medicine and Health, Leeds Institute of Health Sciences<br>Green, Sophie M. C.; University of Leeds Faculty of Medicine and Health, Leeds Institute of Health Sciences<br>MacKenzie, Mairead; Independent Cancer Patients' Voice<br>Taylor, David; University College London, Pharmacy<br>Smith, Samuel; University of Leeds Faculty of Medicine and Health, Leeds Institute of Health Sciences
Keywords:	PREVENTIVE MEDICINE, Behavior, Gastrointestinal tumours < ONCOLOGY, Decision Making

SCHOLARONE™
Manuscripts

I, the Submitting Author has the right to grant and does grant on behalf of all authors of the Work (as defined in the below author licence), an exclusive licence and/or a non-exclusive licence for contributions from authors who are: i) UK Crown employees; ii) where BMJ has agreed a CC-BY licence shall apply, and/or iii) in accordance with the terms applicable for US Federal Government officers or employees acting as part of their official duties; on a worldwide, perpetual, irrevocable, royalty-free basis to BMJ Publishing Group Ltd ("BMJ") its licensees and where the relevant Journal is co-owned by BMJ to the co-owners of the Journal, to publish the Work in this journal and any other BMJ products and to exploit all rights, as set out in our licence.

The Submitting Author accepts and understands that any supply made under these terms is made by BMJ to the Submitting Author unless you are acting as an employee on behalf of your employer or a postgraduate student of an affiliated institution which is paying any applicable article publishing charge ("APC") for Open Access articles. Where the Submitting Author wishes to make the Work available on an Open Access basis (and intends to pay the relevant APC), the terms of reuse of such Open Access shall be governed by a Creative Commons licence – details of these licences and which Creative Commons licence will apply to this Work are set out in our licence referred to above.

Other than as permitted in any relevant BMJ Author's Self Archiving Policies, I confirm this Work has not been accepted for publication elsewhere, is not being considered for publication elsewhere and does not duplicate material already published. I confirm all authors consent to publication of this Work and authorise the granting of this licence.

For peer review only

Title: Acceptability of aspirin for cancer preventive therapy: a mixed methods study exploring the views of the UK general population

Authors: Kelly E. Lloyd[1], Louise H. Hall[1], Lucy Ziegler[1], Robbie Foy[1], Sophie M. C. Green[1], Mairead MacKenzie[2], David G. Taylor[3], Samuel G. Smith[1]

Affiliations:

1. Leeds Institute of Health Sciences, University of Leeds, Leeds, UK
2. Independent Cancer Patients' Voice, London, UK
3. School of Pharmacy, University College London, London, UK

Corresponding author: Kelly E. Lloyd, Leeds Institute of Health Science, University of Leeds, Clarendon Way, Leeds, LS2 9NL, UK. Email: k.e.lloyd@leeds.ac.uk. ORCID: 0000-0002-0420-2342.

Key words: Preventive therapy; chemoprevention; cancer prevention; decision-making; aspirin; NSAID

Manuscript word count: 3,507 / 4,000

Abstract word count: 292/ 300

For peer review only

For peer review only - http://bmjopen.bmj.com/site/about/guidelines.xhtml

ABSTRACT

Objectives: Aspirin could be offered for colorectal cancer prevention for the UK general population, following the Australian national guidance for adults aged 50-70. To ensure the views of the general population are considered in future guidance, we explored public perceptions of aspirin for preventive therapy.

Design: We conducted an online survey to investigate aspirin use, and awareness of aspirin for cancer prevention among the UK general population. We conducted semi-structured interviews with a sub-sample of survey respondents to explore participants' acceptability towards aspirin for cancer preventive therapy. We analysed the interview data using reflexive thematic analysis, and mapped the themes onto the Theoretical Domains Framework, and the Necessity and Concerns Framework.

Setting: Online survey and remote interviews.

Participants: We recruited 400 UK respondents aged 50-70 through a market research company to the survey. We purposefully sampled, recruited and interviewed 20 survey respondents.

Results: In the survey, 19.0% (76/400) of respondents were aware aspirin can be used to prevent cancer. Among those who had previously taken aspirin, 1.9% (4/216) had taken it for cancer prevention. The interviews generated three themes: 1) Perceived necessity of aspirin; 2) Concerns about side-effects; and 3) Preferred information sources. Participants with a personal or family history of cancer were more likely to perceive aspirin as necessary for cancer prevention. Concerns regarding aspirin side-effects were common. Many described wanting guidance and advice on aspirin to be communicated from sources perceived as trustworthy, such as healthcare professionals.

Conclusions: Among the general population, those with a personal or family history of cancer may be more receptive towards taking aspirin for preventive therapy. Future policies and campaigns recommending aspirin may be of particular interest to these groups. Concerns about the side-effects were common, highlighting the need to support informed decisions on aspirin.

For peer review only - http://bmjopen.bmj.com/site/about/guidelines.xhtml

Strengths and limitations of this study

- The online survey collected unique data on aspirin use and reasons for taking aspirin among the UK general population aged 50 to 70.
- The interviews offered the opportunity to explore the potential barriers and facilitators to taking aspirin for cancer prevention among the general public.
- Self-selection to the survey and interviews may have resulted in recruiting participants with stronger views on aspirin than the wider UK population.

For peer review only

For peer review only - http://bmjopen.bmj.com/site/about/guidelines.xhtml

INTRODUCTION

Colorectal cancer is one of the most common cancers worldwide, with an estimated two million cases and nearly one million deaths from the disease globally in 2020 (1). There is increasing interest in the pharmacological prevention of cancer (2), including aspirin to prevent colorectal cancer (3). A pooled analysis of 423,495 people from two cohort studies found daily aspirin use was associated with a 15% reduced risk of colorectal cancer (hazard ratio: 0.85, 95% CI=0.80-0.89) (4). Furthermore, a meta-analysis of four randomised controlled trials investigating aspirin for vascular disease prevention found aspirin was associated with reduced colon cancer incidence (hazard ratio: 0.76, 95% CI=0.60-0.96) (5). Studies have investigated use of aspirin for preventing other cancers, however the evidence is more limited (6).

Aspirin is often recommended for people at higher risk of developing colorectal cancer. Australian national guidance (7), and the United Kingdom (UK) National Institute for Health and Care Excellence (guidance NG151) recommend aspirin for colorectal cancer prevention among people with Lynch syndrome (8). The guidance does not state a recommended dose, but 150-300mg is commonly used in practice. Australian guidance also recommends considering 100-300mg daily aspirin for those in the general population aged 50 to 70 to prevent colorectal cancer (7). There is no current equivalent national guidance for the public in the UK or United States (US).

Decisions on whether to use aspirin involve consideration of potential benefits and side-effects, such as an increased risk of gastrointestinal bleeding (9). For individuals at population risk of colorectal cancer, regular aspirin use between 75mg to 325mg appears to have a favourable benefit-harm profile (6), although the risk of side-effects increases substantially after age 70 (6, 10, 11). Current use and acceptability of aspirin for colorectal cancer prevention among the UK public is unknown (12). It is likely though that a proportion of the public have experience taking aspirin (13), such as for pain relief, or cardiovascular disease risk reduction (14).

Most research examining barriers to using preventive therapy has focused on pharmacological prevention of breast cancer among women at higher risk of the disease. Barriers to use include concerns about side-effects (15-18), and perceptions of the medication as a 'cancer drug' (15). Research has also explored the views of people with Lynch syndrome on aspirin for colorectal cancer prevention (19). Several barriers to taking aspirin were observed, including concerns regarding aspirin's side-effects, and confusion on the recommended dose. It is important to explore the views of the general population on aspirin for cancer prevention, as there are likely to be similar and different barriers to initiating aspirin compared with those at higher risk. Australian cross-sectional research has observed moderately high acceptance (>70%) for taking aspirin regularly for colorectal

For peer review only - http://bmjopen.bmj.com/site/about/guidelines.xhtml

cancer prevention among the general population (20). However, the study did not explore participants' motivators and barriers towards the use of aspirin.

The potential impact of using aspirin for cancer prevention in the wider population will depend on acceptability as well as effectiveness, and an understanding of the barriers to implementation. Public perceptions of aspirin for cancer preventive therapy should be explored to inform both clinical guideline development (21), and support informed decision-making (22). In this study, we recruited people from the UK public to investigate their aspirin use and associated knowledge. In addition, we explored the potential facilitators and barriers towards taking aspirin for colorectal cancer prevention among a sub-sample of survey respondents.

METHODS

Design

We carried out a mixed methods study. We conducted an online survey for two reasons: 1) to recruit people from the UK general population to an interview; 2) to collect initial data on participants' prior use of aspirin and their knowledge on using aspirin for cancer prevention. Following the survey, we conducted semi-structured, one-to-one interviews with a sub-sample of survey participants to explore their perceptions of aspirin for colorectal cancer prevention. We pre-registered the methods and analysis plan (https://doi.org/10.17605/OSF.IO/3EFG7). Study reporting was guided by the Standards for Reporting Qualitative Research (SRQR) checklist (23).

Participants and recruitment

We hosted the online survey on Qualtrics, and recruitment was advertised through a market research company (Dynata). We recruited people from the UK public between the ages of 50 to 70, as the benefits of prophylactic aspirin use are estimated to be greater than the risks of side-effects within this age range (6). At the end of the survey, we asked respondents for contact details if they wished to take part in a follow-up interview. All interviewees received £25 from Dynata. One author (KEL) recruited participants until data saturation was considered to have been achieved, following an established method (24). After 10 interviews, we assessed for data saturation and stopped recruitment once three subsequent interviews had been conducted and no new themes were identified (24). For example, recruitment would stop after participant 13, if no new themes were observed from participants 11, 12, and 13.

For peer review only - http://bmjopen.bmj.com/site/about/guidelines.xhtml

Survey measures

Aspirin use

We asked participants whether they had ever taken aspirin. Those who answered yes were asked if they took aspirin regularly (i.e. most days or every day) and their reasons for taking aspirin, such as for pain relief, cancer prevention, or cardiovascular disease prevention (Supplementary Materials 1).

Knowledge

We asked participants if they were aware prior to the survey that aspirin can be used to reduce the risk of developing certain cancers. We also asked whether a healthcare provider had previously discussed with them about taking aspirin to prevent colorectal and other cancers.

Characteristics of the sample

We collected data on participant characteristics, including any previous cancer diagnoses, gender, age, ethnicity, and highest educational or professional qualification obtained.

Interview schedule

We developed the interview schedule based on the Theoretical Domains Framework (TDF; version 2) (25), which is a framework derived from multiple behaviour change theories (Supplementary Materials 2). Each of the 14 framework domains describes a factor that could influence individual behaviour when implementing new clinical practices. The TDF domains cover both internal factors, such as a person's knowledge and emotions, and external factors, such as social influences and available resources.

We conducted semi-structured interviews with flexibility to the order of questions, and improvised follow-up questions. At the beginning of all interviews, participants were informed that aspirin is currently only recommended in the UK for people at higher risk of colorectal cancer due to a genetic syndrome, but that there is potential for wider recommended use in the future.

Patient and public involvement

A public representative (MM) reviewed the draft interview schedule to ensure the questions were comprehensible to participants.

Data collection and analysis

One author (KEL) analysed the survey data in R Studio (R version 4.2.1), with findings presented in proportions and frequencies. One author (KEL), with previous experience in collecting and analysing qualitative data, conducted all interviews by video call or telephone. Interviews took place from July to August 2022. Interviews were audio-recorded, transcribed verbatim, and pseudonymised using initials to replace participants' names.

For peer review only - http://bmjopen.bmj.com/site/about/guidelines.xhtml

The interview data was analysed in two stages, which is recommended for the TDF to optimise its use in qualitative research (26), and can identify themes not captured by a theoretical framework (26, 27). The interview transcripts were coded and analysed using reflexive thematic analysis (28, 29). The developed themes were then mapped onto the TDF domains (25). During analysis, we found the Necessity-Concerns Framework to be an additional useful framework for guiding our analytic process (30). The framework specifies that people consider their treatment necessity beliefs against their concerns when making decisions on medication (31, 32). One author (KEL) coded and analysed all transcripts, and two authors (SGS, SMCG) double coded a proportion of interviews. Themes were discussed and finalised collaboratively among the three authors. One author (KEL) mapped the themes onto the domains in the TDF (version 2), which was reviewed by co-authors. All transcripts were managed in NVivo (version 1.6.1) and Microsoft Word.

RESULTS

Survey findings

Four hundred people participated in the survey (Table 1). The mean age of the sample was 60.8 years (SD=5.7). Most of the sample were men (213; 53.3%), educated below degree level (251; 62.8%), and were white British or Irish (371; 92.8%). Seventy-six (19.0%) were aware prior to the survey that aspirin can be used to reduce the risk of developing certain cancers, and 15 (3.8%) had previously discussed aspirin for this purpose with a healthcare professional.

Most participants (216; 54.0%) had taken aspirin at least once. Among the 216 previous aspirin users, most had taken it for pain relief (150; 69.4%). Fewer had taken aspirin for prevention of cardiovascular disease (61; 28.2%) and cancer (4; 1.9%). One person (0.5%) had taken aspirin as part of a trial. Among the 216 previous aspirin users, 59 (27.3%) reported using aspirin regularly, defined as most days or every day. Most of the 59 regular aspirin users took it for cardiovascular disease prevention (45; 76.3%). Other reasons for regularly taking aspirin were pain relief (11/59, 18.6%), and cancer prevention (3/59; 5.1%).

Table 1. Characteristics of the survey respondents, recruited from the UK general population (n=400)

	***n* (%)**
**Age**	
50-55	88 (22.0%)
56-60	106 (26.5%)
61-65	103 (25.8%)

For peer review only - http://bmjopen.bmj.com/site/about/guidelines.xhtml

66-70	103 (25.8%)
**Gender**	
Women	186 (46.5%)
Men	213 (53.3%)
Another identity	1 (0.3%)
**Ethnicity**	
White British or Irish	371 (92.8%)
White Gypsy or Irish Traveller	1 (0.3%)
Any other White background	12 (3.0%)
Asian or Asian British	9 (2.3%)
Mixed White and Asian	1 (0.3%)
Arab or Arab British	2 (0.5%)
Black/ African/ Caribbean background / mixed background	2 (0.5%)
Any other ethnic background / mixed background	2 (0.5%)
**Education**	
Degree level and above	149 (37.3%)
Below degree level	251 (62.8%)
**Previously diagnosed with cancer**	
Yes	46 (11.5%)
No	354 (88.5%)
**Among those 'Yes' to cancer (*n*=46), which cancer(s)**	
Prostate	11 (23.9%)
Breast	10 (21.7%)
Skin	6 (13.0%)
Colorectal	5 (10.9%)
Other cancers	14 (30.4%)

Note: Proportions may not compute to 100% due to rounding.

For peer review only - http://bmjopen.bmj.com/site/about/guidelines.xhtml

Interview findings

Two hundred and two (50.5%) survey respondents expressed interest in a follow-up interview. We invited participants by email to be interviewed until we judged data saturation was reached. We purposefully sampled participants from different demographic groups (e.g. gender), and a balance of current and never aspirin users. In batches of 5-10 invitations, we invited 53 survey respondents to be interviewed, and 20 (37.7%) responded and were interviewed (Table 2). Interview duration ranged between 15 to 30 minutes. We identified three overarching themes, and interview findings were mapped onto the TDF (Table 3).

Table 2. Characteristics of the general public interview respondents (n = 20)

	***n* (%)**
**Age**	
50-55	3 (15.0%)
56-60	7 (35.0%)
61-65	5 (25.0%)
66-70	5 (25.0%)
**Gender**	
Women	12 (60.0%)
Men	8 (40.0%)
**Ethnicity**	
White British or Irish	17 (85.0%)
Asian or Asian British	3 (15.0%)
**Country in the UK**	
England	18 (90.0%)
Scotland	1 (5.0%)
Northern Ireland	1 (5.0%)
**Previously diagnosed with cancer**	
Yes	6 (30.0%)
No	14 (70.0%)

For peer review only

For peer review only - http://bmjopen.bmj.com/site/about/guidelines.xhtml

Table 3. The themes, and corresponding facilitators, barriers, and domains within the Theoretical Domains Framework (TDF; version 2)

Themes	Potential facilitators to the use of aspirin for preventive therapy	Potential barriers to the use of aspirin for preventive therapy	Main TDF domain(s)
Perceived necessity of aspirin	Those who perceive themselves at higher risk of cancer because they have a personal or family history of the disease.	Those who perceive themselves at lower risk of cancer because they do not have a personal or family history of the disease.	Beliefs about consequences
Concerns about side-effects	Low concerns regarding the side-effects of aspirin because it is a well-known over-the-counter medication for pain relief.	High concerns about using daily aspirin because of the side-effects, such as increased risk of gastrointestinal bleeding.<br>Concerns about taking aspirin daily at doses above 75mg.	Beliefs about consequences
Preferred information sources	Wanting information on aspirin to come from trusted online sources of information, such as the NHS and UK cancer charities (e.g. Cancer Research UK).<br>Wanting information on aspirin to come from a medical professional.	Current lack of a recommendation to support the use of aspirin for colorectal cancer prevention among the UK general public.	Environmental context and resources

Note. Table adapted from Burgess et al. (33).

For peer review only - http://bmjopen.bmj.com/site/about/guidelines.xhtml

Perceived necessity of aspirin

Did not perceive a need for aspirin

Participant beliefs varied about the perceived necessity of taking aspirin for colorectal cancer prevention. Several people perceived themselves to be at low risk of the disease, typically because they had no family history or personal history of cancer. Often, these participants described how they would only consider using aspirin for cancer prevention if a medical doctor assessed them to be at higher risk, or if they were diagnosed with colorectal cancer.

> *"If someone said to me I was high risk and aspirin was the, was a possible option, I would take it." (J.J., 56 years old, woman).*

> *"Yeah, well I think probably if I have had bowel cancer and I was in recovery […] then I probably would take [aspirin]." (W.G., 69 years old, man).*

Several participants were averse to using daily medication in general, unless deemed highly necessary for their health.

> *"I would only take [medication] if it was really necessary." (A.G., 69 years old, woman).*

> *"On the whole I don't like taking pills, […]. Unless there was very definite proof that not taking [aspirin] would lead to bowel cancer." (W.G., 69 years old, man).*

A few participants perceived other lifestyle changes, such as diet, as more effective and necessary than taking medication to prevent cancer.

> *"I think [colorectal cancer] can be broadly governed by diet, fibre, etc." (E.Y., 60 years old, man).*

Perceived a necessity for aspirin

A number of participants discussed having previously been diagnosed with cancer (e.g., breast, colorectal, prostate), which in turn increased their interest in using aspirin, and their feelings on the importance of cancer prevention.

> *"If I saw the GP yesterday […] 'oh would you like to take aspirin as a preventative for bowel cancer?' I would say yes, particularly now that I've been diagnosed with, with breast cancer." (I.N., 64 years old, woman).*

Several participants appeared more inclined to use aspirin because friends or family members had been diagnosed with cancer. All three interviewees who currently used or had previously used aspirin for cancer prevention had started because of a family history of cancer.

For peer review only - http://bmjopen.bmj.com/site/about/guidelines.xhtml

"A few years ago I heard an article on [a public service radio station] and there was two eminent doctors, and they were talking about the possibility that aspirin prevented not just bowel cancer but other cancers. […] That triggered me to start taking it, my father died of cancer and my sister also died of cancer." (H.B., 69 years old, man).

Concerns about side-effects

Expressed high concerns about aspirin

Many participants expressed concerns about the side-effects of daily aspirin use, such as increased risk of gastrointestinal bleeding. In a Lynch syndrome population, aspirin is often prescribed in clinical practice at a daily dose of 150-300mg (8). However, several participants expressed feeling more comfortable using a lower dose of aspirin (e.g. 75mg), due to concerns about side-effects.

"Because [150-300mg] seems to be a lot. […], but 75mg, a small dose, I would feel comfortable with that." (S.S., 70 years old, woman).

Several participants' concerns regarding aspirin appeared to be related to the perceived harm of taking any long-term medication. These participants felt that taking daily medication would make them go from *"being a healthy person to a potentially unhealthy person" (R.C., 58 years old, man).* Most participants wanted further information on the side-effects of aspirin before they would consider initiating the medication. Participants often wanted to know if the benefits of aspirin for preventive therapy substantially outweighed the side-effects.

"I probably would like to know more about what effects it would have on my body, what damage it could do to my heart, or other organs." (K.H., 60 years old, woman).

Did not have concerns about aspirin

Despite widespread concerns about aspirin, these views were not universally held among participants. Aspirin was a familiar drug to many, which lowered some concerns about side-effects.

"Because it's been well known to me that [aspirin] can help prevent heart problems and that's kind of been around for decades […] So I think knowing that and not hearing of anything happening to anybody then that's very reassuring for me." (R.L., 57 years old, woman).

Some participants expressed low concerns about aspirin because of their own or family members previous experiences using the medication without encountering side-effects.

For peer review only - http://bmjopen.bmj.com/site/about/guidelines.xhtml

"No, no I don't [have concerns about the side-effects], because you know whenever I've taken it, […] I don't take them every day but my mother had no side-effects whatsoever." (I.N., 64 years old, woman).

Preferred information sources

Participants discussed their preferred source of information on the use of aspirin for colorectal cancer prevention. Many participants wanted the information on aspirin to come from trusted online sources, such as the National Health Service (NHS) and UK cancer charities (e.g. Cancer Research UK).

"Cancer Research would probably be, or Macmillan, it would be a trusted website that […] I would look at." (K.H., 60 years old, woman).

Other important information sources discussed were medical professionals. Several participants wanted the information on aspirin for cancer prevention to come from their GP, while others preferred speaking to a cancer specialist. In most cases, participants felt that they would not initiate aspirin for preventive therapy without a doctor's recommendation.

"Well certainly I think I would first and primarily go to the GP." (A.N., 68 years old, woman).

"Maybe not a GP but somebody who was specialised in that area, maybe a gastroenterologist […] I wouldn't automatically [take aspirin] because I wouldn't have enough information to warrant what else it could do to me." (K.H., 60 years old, woman).

Several people expressed positive views towards speaking to a pharmacist about aspirin, often because they had previously had positive experiences consulting their pharmacists on other medical issues. However, not all were comfortable with this approach, and only wanted to speak with a medical doctor about aspirin.

"Well I think my doctor is the trustworthy cause, but also my pharmacy is really good." (A.G., 69 years old, woman).

"I've always gone to GP, I've never discussed anything with a pharmacist ever […] I mean they're giving a lot of powers to the pharmacist, but I don't feel comfortable talking to a pharmacist." (L.L., 62 years old, man).

For peer review only - http://bmjopen.bmj.com/site/about/guidelines.xhtml

Official UK guidance recommending aspirin for cancer prevention for the general public was an important factor to some participants. However, this was only mentioned by a small number of people as a potential barrier to using aspirin.

> *"I'd be happy if [aspirin] was recommended though, but I'd wait till it was recommended, I wouldn't jump on things too quickly." (F.L., 52 years old, man).*

DISCUSSION

This study of the UK general population found low reported current use of aspirin for cancer prevention among survey respondents, and varying acceptability towards taking aspirin for this purpose across interviewees. In the interviews, those with a personal or family history of cancer were more receptive to taking aspirin whilst those who considered themselves at lower risk of colorectal cancer, or cancer in general, expressed more resistance. Public perceptions towards taking aspirin for cancer prevention should be considered in any future guidance recommending aspirin outside of a Lynch syndrome population.

Most survey respondents had taken aspirin, often for pain relief. People's perceptions of aspirin as a well-known medicine may support its use for prevention, as several participants interviewed held positive views towards aspirin for this reason. However, many had concerns about side-effects, highlighting the need to support informed choices about taking medication. Awareness of aspirin for cancer prevention among survey respondents was low, and only a small percentage took aspirin for prevention. The interview findings suggest family history of cancer was an important motivator to taking aspirin among those at population risk.

We considered our qualitative findings in relation to the 14 domains in the TDF, and identified two main domains (25). These domains were the 'Beliefs about the consequences' of using aspirin, and the potential 'Environmental context and resources' which would aid in implementing aspirin for the public. Participant beliefs about the perceived necessity for taking aspirin and concerns about side-effects were particularly important, and relate to the Necessity-Concerns Framework (30). Previous evidence has found those who report low necessity for a medication and high concerns on the side-effects are less likely to initiate and adhere to a range of different medications (34-37). Similarly, a UK prospective study found women at higher risk of breast cancer with low concerns about side-effects were significantly more likely to initiate preventive medication (38).

While the interview findings suggest a relationship between perceived cancer risk and uptake of aspirin, a large population survey is warranted to investigate these potentially motivating factors further. Although minimal previous research has been conducted in this area (12), surveys in the US

For peer review only - http://bmjopen.bmj.com/site/about/guidelines.xhtml

and Australia have observed mixed findings on a relationship between cancer risk and interest in aspirin for colorectal cancer prevention (20, 39). Interventions aiming to support informed uptake of aspirin for preventive therapy should consider targeting people's beliefs regarding the side-effects and benefits of taking the medication. There is also scope for research to explore the relationship between the Necessity-Concerns Framework and adherence to aspirin.

Guidance recommending aspirin for preventive therapy among the public should consider how the information is communicated, as several participants want the advice to come from a healthcare professional. People who have previously had cancer or a precursor to cancer may be particularly receptive to taking aspirin for preventive therapy. There is trial and observational evidence supporting the use of aspirin among people with colorectal adenomas (40-46), and this may be an appropriate group for policy makers to target if the harm-benefit profile is deemed sufficient. While the evidence for using aspirin for secondary cancer prevention is less developed (47), the ongoing Add-Aspirin trial is investigating the effectiveness of regular aspirin for patients with non-metastatic colorectal, breast, gastro-oesophageal, and prostate cancer (48, 49). Our findings suggest such groups may be particularly receptive to receiving a recommendation to use aspirin for preventive therapy.

This study had limitations. We recruited a moderate sample size of 400 respondents to the survey, therefore the findings should be generalised to the wider UK population with caution. In some cases, weighting approaches can be employed with the aim to weight participants' responses to represent the target population (50). However, we concluded that weighting was inappropriate for our survey as several demographic (e.g. ethnicity) categories contained little to no data, which can lead to over or underrepresenting responses for these population groups (51). Self-selection to the survey and interviews may have also resulted in recruiting people with stronger views on the topic than those in the general population (52).

For the interviews, most participants were white, with only three people recruited from an Asian background. Further research is warranted to explore the views of people from different ethnic minority groups on the use of aspirin for cancer preventive therapy. There are likely to be specific barriers among some ethnic minority groups. For example, research has observed South Asian respondents to view cancer as a taboo subject (53, 54), and have discussed the stigma attached to taking long-term medications (55-57), which could reduce uptake of cancer preventive therapy.

CONCLUSION

People with personal or family history of cancer were particularly receptive towards aspirin for colorectal cancer prevention. Future guidance recommending aspirin for cancer prevention may be

For peer review only - http://bmjopen.bmj.com/site/about/guidelines.xhtml

of particular interest to these groups. Concerns about the side-effects of aspirin were common across the sample, highlighting the need to support informed decisions on the medication. Guidance and advice recommending aspirin should be communicated from sources perceived as trustworthy by the public, such as healthcare professionals.

For peer review only

For peer review only - http://bmjopen.bmj.com/site/about/guidelines.xhtml

DECLARATIONS

Acknowledgements: We thank all the participants who took part in the study. We also acknowledge the AsCaP senior executive board and committee members.

Ethical approval: Ethical approval was awarded by the University of Leeds School of Medicine Research Ethics Committee (MREC 19-091). Informed consent was obtained from all individual participants included in this study.

Competing interests: Robbie Foy is a member of the NICE Implementation Strategy Group. All other authors have no competing interests to declare that are relevant to the content of this article.

Funding: This work is fully funded by the Aspirin for Cancer Prevention AsCaP Group CRUK Grant Code: **A24991,** Senior Executive Board Prof. J Burn, Prof. A.T Chan, Prof. J Cuzick, Dr. B Nedjai, Prof. Ruth Langley. During the study, KEL was supported by an Economic and Social Research Council studentship [grant number ES/P000745/1]. This report is independent research supported by the National Institute for Health Research NIHR Advanced Fellowship, Professor Samuel Smith, [grant number NIHR300588]. SGS also acknowledges funding support from a Yorkshire Cancer Research University Academic Fellowship. The funders had no role in study design, data collection and analysis, decision to publish, or preparation of the manuscript.

Data sharing statement: The survey data is available in a public, open access repository. The survey dataset and analysis scripts are publicly available in the Research Data Leeds Repository: https://doi.org/10.5518/1379. The qualitative data is available upon reasonable request.

Author contributions: Conceptualisation: K.E.L, S.G.S, R.F, L.H.H, and L.Z. Methodology: K.E.L, S.G.S, R.F, L.H.H, L.Z, D.G.T, and M.M. Supervision: S.G.S, R.F, L.H.H, and L.Z. Funding acquisition: S.G.S, D.G.T, and M.M. Investigation: K.E.L. Formal analysis: K.E.L, S.M.C.G, and S.G.S. Writing – original draft: K.E.L. Writing – review and editing: all authors.

Aspirin for Cancer Prevention AsCaP Steering Committee Members:

Professor Jack Cuzick, Queen Mary University of London. Chair

Professor Frances Balkwill, Queen Mary University of London

Professor Tim Bishop, University of Leeds

Professor Sir John Burn, Newcastle University

Professor Andrew T. Chan, Harvard School of Medicine

For peer review only - http://bmjopen.bmj.com/site/about/guidelines.xhtml

For peer review only

Dr Colin Crooks, University of Nottingham

Professor Chris Hawkey, University of Nottingham

Professor Ruth Langley, University College London

Ms Mairead McKenzie, Independent Cancer Patients' Voice

Dr Belinda Nedjai, Queen Mary University of London

Professor Paola Patrignani, Università "G. d'Annunzio" di Chieti-Pescara

Professor Carlo Patrono, Catholic University of the Sacred Heart, Rome

Dr Bianca Rocca, Catholic University of the Sacred Heart, Rome

Professor Samuel Smith, University of Leeds

Dr Laura Greaves, Newcastle University

For peer review only

For peer review only - http://bmjopen.bmj.com/site/about/guidelines.xhtml

REFERENCES

1. Xi Y, Xu P. Global colorectal cancer burden in 2020 and projections to 2040. Transl Oncol. 2021;14 (10):101174.

2. Benetou V, Lagiou A, Lagiou P. Chemoprevention of cancer: current evidence and future prospects. F1000Res. 2015;4 (F1000 Faculty Rev):916.

3. Katona BW, Weiss JM. Chemoprevention of Colorectal Cancer. Gastroenterology. 2020;158 (2):368-88.

4. Hurwitz LM, Michels KA, Cook MB, Pfeiffer RM, Trabert B. Associations between daily aspirin use and cancer risk across strata of major cancer risk factors in two large U.S. cohorts. Cancer Causes Control. 2021;32 (1):57-65.

5. Rothwell PM, Wilson M, Elwin CE, Norrving B, Algra A, Warlow CP, et al. Long-term effect of aspirin on colorectal cancer incidence and mortality: 20-year follow-up of five randomised trials. Lancet. 2010;376 (9754):1741-50.

6. Cuzick J, Thorat MA, Bosetti C, Brown PH, Burn J, Cook NR, et al. Estimates of benefits and harms of prophylactic use of aspirin in the general population. Ann oncol. 2015;26 (1):47-57.

7. Cancer Council Australia. Chemopreventive candidate agents. [Online]. 2019 [Accessed August 2022]. Available from: https://wiki.cancer.org.au/australia/Clinical_question:Aspirin_for_prevention_of_colorectal_cancer.

8. National Institute for Health and Clinical Excellence (NICE). Colorectal cancer [NG151]. [Online]. 2020. [Accessed August 2022]. Available from: https://www.nice.org.uk/guidance/NG151.

9. Sørensen HT, Mellemkjær L, Blot WJ, Nielsen GL, Steffensen FH, McLaughlin JK, et al. Risk of upper gastrointestinal bleeding associated with use of low-dose aspirin. Am J Gastroenterol. 2000;95 (9):2218-24.

10. McNeil JJ, Wolfe R, Woods RL, Tonkin AM, Donnan GA, Nelson MR, et al. Effect of Aspirin on Cardiovascular Events and Bleeding in the Healthy Elderly. N Engl J Med. 2018;379 (16):1509-18.

11. McNeil JJ, Gibbs P, Orchard SG, Lockery JE, Bernstein WB, Cao Y, et al. Effect of Aspirin on Cancer Incidence and Mortality in Older Adults. J Natl Cancer Inst. 2021;113 (3):258-65.

12. Lloyd KE, Hall LH, King N, Thorneloe RJ, Rodriguez-Lopez R, Ziegler L, et al. Aspirin use for cancer prevention: A systematic review of public, patient and healthcare provider attitudes and adherence behaviours. Prev Med. 2021;154:106872.

For peer review only - http://bmjopen.bmj.com/site/about/guidelines.xhtml

13. Trinder P, Rajaratnam G, Lewis M, Croft P. Prophylactic aspirin use in the adult general population. J Public Health Med. 2003;25 (4):377-80.

14. Campbell CL, Smyth S, Montalescot G, Steinhubl SR. Aspirin dose for the prevention of cardiovascular disease: a systematic review. JAMA. 2007;297 (18):2018-24.

15. Donnelly LS, Evans DG, Wiseman J, Fox J, Greenhalgh R, Affen J, et al. Uptake of tamoxifen in consecutive premenopausal women under surveillance in a high-risk breast cancer clinic. Br J Cancer. 2014;110 (7):1681-7.

16. Heisey R, Pimlott N, Clemons M, Cummings S, Drummond N. Women's views on chemoprevention of breast cancer: qualitative study. Can Fam Physician. 2006;52 (5):624-5.

17. Altschuler A, Somkin CP. Women's Decision Making About Whether or Not to Use Breast Cancer Chemoprevention. Women Health. 2005;41 (2):81-95.

18. Hackett J, Thorneloe R, Side L, Wolf M, Horne R, Cuzick J, et al. Uptake of breast cancer preventive therapy in the UK: results from a multicentre prospective survey and qualitative interviews. Breast Cancer Res Treat. 2018;170 (3):633-40.

19. Lloyd KE, Foy R, Hall LH, Ziegler L, Green SMC, Haider ZF, et al. Barriers and facilitators to using aspirin for preventive therapy: a qualitative study exploring the views and experiences of people with Lynch syndrome and healthcare providers. Hered Cancer Clin Pract. 2022;20 (1):30.

20. Nguyen P, McIntosh J, Bickerstaffe A, Maddumarachchi S, Cummings K-L, Emery JD. Benefits and harms of aspirin to reduce colorectal cancer risk: a cross-sectional study of methods to communicate risk in primary care. Br J Gen Pract. 2019;69(689):e843.

21. Boivin A, Currie K, Fervers B, Gracia J, James M, Marshall C, et al. Patient and public involvement in clinical guidelines: international experiences and future perspectives. Qual Saf Health Care. 2010;19 (5):e22.

22. Stiggelbout AM, Weijden TVd, Wit MPTD, Frosch D, Légaré F, Montori VM, et al. Shared decision making: really putting patients at the centre of healthcare. BMJ. 2012;344:e256.

23. O'Brien BC, Harris IB, Beckman TJ, Reed DA, Cook DA. Standards for Reporting Qualitative Research: a synthesis of recommendations. Acad Med. 2014;89(9).

For peer review only

For peer review only - http://bmjopen.bmj.com/site/about/guidelines.xhtml

24. Francis JJ, Johnston M, Robertson C, Glidewell L, Entwistle V, Eccles MP, et al. What is an adequate sample size? Operationalising data saturation for theory-based interview studies. Psychol Health. 2010;25 (10):1229-45.

25. Atkins L, Francis J, Islam R, O'Connor D, Patey A, Ivers N, et al. A guide to using the Theoretical Domains Framework of behaviour change to investigate implementation problems. Implement Sci. 2017;12 (1):77.

26. McGowan LJ, Powell R, French DP. How can use of the Theoretical Domains Framework be optimized in qualitative research? A rapid systematic review. Br J Health Psychol. 2020;25 (3):677-94.

27. MacFarlane A, O'Reilly-de Brún M. Using a theory-driven conceptual framework in qualitative health research. Qual Health Res. 2012;22 (5):607-18.

28. Braun V, Clarke V. One size fits all? What counts as quality practice in (reflexive) thematic analysis? Qual Res Psychol. 2021;18 (3):328-52.

29. Braun V, Clarke V. Using thematic analysis in psychology. Qual Res Psychol. 2006;3 (2):77-101.

30. Horne R, Weinman J, Hankins M. The beliefs about medicines questionnaire: The development and evaluation of a new method for assessing the cognitive representation of medication. Psychol Health. 1999;14 (1):1-24.

31. Horne R, Chapman SCE, Parham R, Freemantle N, Forbes A, Cooper V. Understanding patients' adherence-related beliefs about medicines prescribed for long-term conditions: a meta-analytic review of the necessity-concerns framework. PLOS ONE. 2013;8 (12):e80633.

32. Phillips LA, Diefenbach MA, Kronish IM, Negron RM, Horowitz CR. The Necessity-Concerns Framework: a multidimensional theory benefits from multidimensional analysis. Ann Behav Med. 2014;48 (1):7-16.

33. Burgess C, Wright AJ, Forster AS, Dodhia H, Miller J, Fuller F, et al. Influences on individuals' decisions to take up the offer of a health check: a qualitative study. Health Expect. 2015;18 (6):2437-48.

34. Clifford S, Barber N, Horne R. Understanding different beliefs held by adherers, unintentional nonadherers, and intentional nonadherers: application of the Necessity–Concerns Framework. J Psychosom Res. 2008;64 (1):41-6.

For peer review only - http://bmjopen.bmj.com/site/about/guidelines.xhtml

35. Horne R, Cooper V, Gellaitry G, Date HL, Fisher M. Patients' perceptions of highly active antiretroviral therapy in relation to treatment uptake and adherence: the utility of the Necessity-Concerns Framework. JAIDS-J ACQ IMM DEF. 2007;45(3).

36. Horne R, Parham R, Driscoll R, Robinson A. Patients' attitudes to medicines and adherence to maintenance treatment in inflammatory bowel disease. Inflamm Bowel Dis. 2009;15 (6):837-44.

37. Clarkesmith DE, Pattison HM, Lip GYH, Lane DA. Educational intervention improves anticoagulation control in atrial fibrillation patients: the TREAT randomised trial. PLOS ONE. 2013;8 (9):e74037.

38. Thorneloe RJ, Horne R, Side L, Wolf MS, Smith SG, Adamson V, et al. Beliefs about medication and uptake of preventive therapy in women at increased risk of breast cancer: results from a multicenter prospective study. Clin Breast Cancer. 2019;19 (1):e116-e26.

39. Jensen JD, Holton AE, Krakow M, Weaver J, Donovan E, Tavtigian S. Colorectal cancer prevention and intentions to use low-dose aspirin: A survey of 1000 U.S. adults aged 40–65. Cancer Epidemiol. 2016;41:99-105.

40. Logan RF, Grainge MJ, Shepherd VC, Armitage NC, Muir KR. Aspirin and folic acid for the prevention of recurrent colorectal adenomas. Gastroenterology. 2008;134 (1):29-38.

41. Sandler RS, Halabi S, Baron JA, Budinger S, Paskett E, Keresztes R, et al. A randomized trial of aspirin to prevent colorectal adenomas in patients with previous colorectal cancer. N Engl J Med. 2003;348 (10):883-90.

42. Baron JA, Cole BF, Sandler RS, Haile RW, Ahnen D, Bresalier R, et al. A randomized trial of aspirin to prevent colorectal adenomas. N Engl J Med. 2003;348 (10):891-9.

43. Benamouzig R, Deyra J, Martin A, Girard B, Jullian E, Piednoir B, et al. Daily soluble aspirin and prevention of colorectal adenoma recurrence: one-year results of the apacc trial. Gastroenterology. 2003;125 (2):328-36.

44. Cole BF, Logan RF, Halabi S, Benamouzig R, Sandler RS, Grainge MJ, et al. Aspirin for the chemoprevention of colorectal adenomas: meta-analysis of the randomized trials. J Natl Cancer Inst. 2009;101 (4):256-66.

45. Grau MV, Sandler RS, McKeown-Eyssen G, Bresalier RS, Haile RW, Barry EL, et al. Nonsteroidal anti-inflammatory drug use after 3 years of aspirin use and colorectal adenoma risk: observational follow-up of a randomized study. J Natl Cancer Inst. 2009;101 (4):267-76.

For peer review only - http://bmjopen.bmj.com/site/about/guidelines.xhtml

46. Chudy-Onwugaje K, Huang W-Y, Su LJ, Purdue MP, Johnson CC, Wang L, et al. Aspirin, ibuprofen, and reduced risk of advanced colorectal adenoma incidence and recurrence and colorectal cancer in the PLCO Cancer Screening Trial. Cancer. 2021;127 (17):3145-55.

47. Langley RE, Burdett S, Tierney JF, Cafferty F, Parmar MKB, Venning G. Aspirin and cancer: has aspirin been overlooked as an adjuvant therapy? Br J Cancer. 2011;105 (8):1107-13.

48. Coyle C, Cafferty FH, Rowley S, MacKenzie M, Berkman L, Gupta S, et al. ADD-ASPIRIN: A phase III, double-blind, placebo controlled, randomised trial assessing the effects of aspirin on disease recurrence and survival after primary therapy in common non-metastatic solid tumours. Contemp clin trials. 2016;51:56-64.

49. Joharatnam-Hogan N, Cafferty F, Hubner R, Swinson D, Sothi S, Gupta K, et al. Aspirin as an adjuvant treatment for cancer: feasibility results from the Add-Aspirin randomised trial. Lancet Gastroenterol Hepatol. 2019;4 (11):854-62.

50. Kalton G, Flores-Cervantes I. Weighting methods. J Off Stat. 2003;19 (2):81.

51. Vaske JJ, Jacobs MH, Sijtsma MT, Beaman J. Can weighting compensate for sampling issues in internet surveys? Hum Dimens Wildl. 2011;16 (3):200-15.

52. Comley P, Beaumont J. Online market research: Methods, benefits and issues — Part 1. J Direct Data Digit Mark Pract. 2011;12(4):315-27.

53. Randhawa G, Owens A. The meanings of cancer and perceptions of cancer services among South Asians in Luton, UK. Br J Cancer. 2004;91 (1):62-8.

54. Karbani G, Lim JN, Hewison J, Atkin K, Horgan K, Lansdown M, et al. Culture, attitude and knowledge about breast cancer and preventive measures: a qualitative study of South Asian breast cancer patients in the UK. Asian Pac J Cancer Prev. 2011;12 (6):1619-26.

55. Kumar K, Greenfield S, Raza K, Gill P, Stack R. Understanding adherence-related beliefs about medicine amongst patients of South Asian origin with diabetes and cardiovascular disease patients: a qualitative synthesis. BMC Endocr Disord. 2016;16 (1):24.

56. Ens TA, Seneviratne CC, Jones C, King-Shier KM. Factors influencing medication adherence in South Asian people with cardiac disorders: an ethnographic study. Int J Nurs Stud. 2014;51 (11):1472-81.

For peer review only

For peer review only - http://bmjopen.bmj.com/site/about/guidelines.xhtml

57. Kamath DY, Bhuvana K, Salazar LJ, Varghese K, Kamath A, Idiculla J, et al. A qualitative, grounded theory exploration of the determinants of self-care behavior among Indian patients with a lived experience of chronic heart failure. PLOS ONE. 2021;16 (1):e0245659.

For peer review only

For peer review only - http://bmjopen.bmj.com/site/about/guidelines.xhtml

Supplementary Materials 1: Short survey on aspirin use for cancer prevention among the general public

1. Do you ever take aspirin?
   a. Yes
   b. No

2. Do you take aspirin regularly (i.e. most days or every day)?
   a. Yes
   b. No
   c. N/A – do not take aspirin

3. What is the main reason you take aspirin?
   a. Pain relief
   b. Prevention of cardiovascular disease (i.e. heart and circulatory disease)
   c. Prevention of cancer
   d. Other reason, please specify _______________
   e. N/A – do not take aspirin

4. Prior to completing this survey, were you aware that aspirin could reduce the risk of developing certain types of cancers?
   a. Yes
   b. No

5. Has your doctor or any other healthcare professional talked to you about how aspirin may lower your risk of developing bowel cancer or any other cancers?
   a. Yes
   b. No

6. Have you previously been diagnosed with cancer?
   a. Yes, please specify which cancer(s): ________________
   b. No

7. How would you describe your gender?
   a. Male
   b. Female
   c. Non-binary
   d. Different identity
   e. Prefer not to say

8. What is your age?

   ____

9. How would you describe your ethnicity?
   a. White - English/ Welsh/ Scottish/ Northern Irish/ British
   b. White - Irish

For peer review only

c. White - Gypsy or Irish Traveller
d. Any other White background
e. Mixed - White and Black Caribbean
f. Mixed - White and Black African
g. Mixed - White and Asian
h. Any other mixed/ multiple ethnic background
i. Asian or Asian British - Indian
j. Asian or Asian British - Pakistani
k. Asian or Asian British - Bangladeshi
l. Asian or Asian British - Chinese
m. Any other Asian background
n. Black or Black British - African
o. Black or Black British - Caribbean
p. Any other Black/ African/ Caribbean background
q. Arab or Arab British
r. Any other ethnic group

10. What is the highest level of educational or professional qualification you have obtained?
    a. GCSE/ O-level/ CSE
    b. Vocational qualifications (e.g. NVQ1+2)
    c. A-Level or equivalent (e.g. NVQ3)
    d. Bachelor's degree or equivalent (e.g. NVQ4)
    e. Masters/ PhD or equivalent
    f. Other qualifications
    g. No formal qualifications

Thank you for taking part in the survey study. If you are interested in taking part further, we are looking for people to take part in a follow-up interview study to explore their thoughts on the use of aspirin for bowel cancer prevention. The interviews will take between 30 to 60 minutes to complete and will be take place either over the telephone or video call, depending upon your preference. You will receive £25 for taking part in the follow-up interview study. You do not need to use aspirin or know anything about this topic to take part.

11. Are you interested in taking part in the follow-up interview study?
    a. Yes, please provide your contact details (e.g. email, phone number) and we will contact you further about the study: ___________________
    b. No

Supplementary Materials 2: Interview schedule for general public interviews

Thank you for agreeing to take part. Just before we start the interview, I'd like to quickly go over some key points about your rights as a participant in this study. It is completely fine if at any point you would like to stop the interview, or if you do not want to answer a question, please just let me know. You can also withdraw from the study at any point and you do not have to provide a reason for why. Just to remind you the interview will be recorded, but anything you say to me today will be kept confidential, and if you are quoted in any of our reports your name will not be used and instead you will be given a pseudonym, which is a fake name. I will also anonymise anything identifiable, like places, you mention as well. After the interview, if you have any further questions about anything we discussed today, please feel free to contact me. I can also provide a debrief sheet at the end with website links with further information on this topic in case you are interested in reading more. Are you happy to continue?

General public interview questions

As you read on the information sheet, the study is aiming to understand what people think about using aspirin for cancer prevention, including the reasons why people may or may not be willing to use it. Aspirin has previously been found to effectively reduce people's risk of bowel cancer in a healthy population sample. At the moment, aspirin is only officially recommended by the NHS for cancer prevention in people who have a genetic condition that makes them higher risk of developing bowel cancer. There is potential in the future though for this medication to be offered more widely to the general public for the purpose of bowel cancer prevention.

While there is the potential benefit of having a reduced likelihood of developing bowel cancer in the future, there are also side-effects to regular aspirin use. The main side-effect is an increased likelihood of experiencing internal bleeding.

I will now move onto the interview questions, which will explore your thoughts on using aspirin regularly for cancer prevention. Just a reminder before we start, you do not need to know anything about the use of aspirin for cancer prevention to answer these questions. We are just interested in hearing your initial thoughts.

For peer review only

Domains	Interview questions
Knowledge<br><br>Enviromental context and resources<br><br>Intentions<br><br>Social influences	What are your initial thoughts on taking aspirin daily for bowel cancer prevention?<br><br>Had you heard of the use of aspirin for cancer prevention before this interview?<br><br>If yes, where and how did you hear about this?<br><br>If no, how would you find such information? Prompts: from what sources? Internet? A healthcare professional?<br><br>Have you ever been encouraged or discouraged by someone to take aspirin regularly for cancer prevention? Prompt: Why was this?
Optimism<br><br>Beliefs about consequences<br><br>Emotions<br><br>Enviromental context and resources<br><br>Skills<br><br>Beliefs about capabilities<br><br>Reinforcement	In your opinion, do you think using aspirin would be a good way to reduce your risk of developing bowel cancer in the future? Prompt: Why do you think this?<br><br>Do you have any concerns about taking aspirin regularly? Prompt: How do you feel about the side-effects?<br><br>What kind of information do you think you would need to help you make a decision on whether to take aspirin regularly for cancer prevention?<br><br>Where would you go to get this information? Prompts: What would your first step be to obtain this information? Internet? Healthcare professionals?<br><br>*[If they do take aspirin for cancer prevention]* Have you previously encountered any problems with trying to get further information on the use of aspirin for cancer prevention? If yes, please describe these problems.<br><br>*[If yes to above question]* Is there anything about this experience that makes you more or less likely to try and take aspirin regularly in the future?
Beliefs about consequences	I mentioned before one of potential side-effects of regular aspirin use can be internal bleeding. There are several risk factors that make a person more likely to experience internal bleeding from regular aspirin use. These include:<br><br>- Active or previous stomach ulcers<br>- Bleeding disorders

For peer review only

	- Being over the age of 65<br>- Uncontrolled hypertension<br>- Previous history of stroke<br>- Abnormal liver or renal function<br>- Experience indigestion on aspirin<br><br>If you had any of these risk factors, your doctor may offer you an additional medication to reduce your risk of internal bleeding.<br><br>What are your thoughts on taking regular aspirin alongside another medication to reduce your risk of bowel cancer?
Goals<br><br>Memory, attention and decision processes	How much of a priority is taking aspirin for cancer prevention to you? Prompts: how high or low a priority is it?<br><br>Are there any other higher priorities? Prompt: Prevention priorities? What are they?

Brief quantitative questions

Thank you so much for your answers so far. Just before we end the interview, there are several brief demographic questions that I would just like to go through with you. If you do not want to answer one of these questions, please just let me know and we can skip it.

1) What is your age?
2) Please describe your gender? Male; Female; Non-binary; Another identity; Prefer not to say.
3) How would you describe your ethnicity? Examples include White British, Indian, Mixed – White and Black Caribbean
4) What country in the UK do you live?
5) Have you previously been diagnosed with cancer?

Debrief

Thank you for all your help with answering my questions. We really appreciate your time and hope that it will be useful in the future when we are trying to support people when making a decision about whether to use aspirin for cancer prevention. Before we end, do you have any questions you would like to ask me?

For peer review only

If you would like more information on the use of aspirin for cancer prevention, I can email you an information sheet with links to website with further information on this topic. If you would like to ask me further questions on the study, please do not hesitate to contact me.

For peer review only

Standards for Reporting Qualitative Research (SRQR)*

http://www.equator-network.org/reporting-guidelines/srqr/

Title and abstract

	Page/line no(s).
**Title** - Concise description of the nature and topic of the study Identifying the study as qualitative or indicating the approach (e.g., ethnography, grounded theory) or data collection methods (e.g., interview, focus group) is recommended	Page 1
**Abstract** - Summary of key elements of the study using the abstract format of the intended publication; typically includes background, purpose, methods, results, and conclusions	Page 2

Introduction

**Problem formulation** - Description and significance of the problem/phenomenon studied; review of relevant theory and empirical work; problem statement	Page 4-5
**Purpose or research question** - Purpose of the study and specific objectives or questions	Page 5

Methods

**Qualitative approach and research paradigm** - Qualitative approach (e.g., ethnography, grounded theory, case study, phenomenology, narrative research) and guiding theory if appropriate; identifying the research paradigm (e.g., postpositivist, constructivist/ interpretivist) is also recommended; rationale**	Page 6-7<br>Theories: Theoretical Domains Framework, and Necessity-Concerns Framework
**Researcher characteristics and reflexivity** - Researchers' characteristics that may influence the research, including personal attributes, qualifications/experience, relationship with participants, assumptions, and/or presuppositions; potential or actual interaction between researchers' characteristics and the research questions, approach, methods, results, and/or transferability	Page 6 – interviewer's previous qualitative experience discussed
**Context** - Setting/site and salient contextual factors; rationale**	Page 5 – online survey<br>Page 6 – telephone or video interview
**Sampling strategy** - How and why research participants, documents, or events were selected; criteria for deciding when no further sampling was necessary (e.g., sampling saturation); rationale**	Page 5 – eligibility and rationale.<br>Page 5 and page 9 – data saturation.
**Ethical issues pertaining to human subjects** - Documentation of approval by an appropriate ethics review board and participant consent, or explanation for lack thereof; other confidentiality and data security issues	Declarations section
**Data collection methods** - Types of data collected; details of data collection procedures including (as appropriate) start and stop dates of data collection and analysis, iterative process, triangulation of sources/methods, and modification of procedures in response to evolving study findings; rationale**	Pages 5 to 7.<br>Interview schedule – supplementary materials.

For peer review only

For peer review only - http://bmjopen.bmj.com/site/about/guidelines.xhtml

**Data collection instruments and technologies** - Description of instruments (e.g., interview guides, questionnaires) and devices (e.g., audio recorders) used for data collection; if/how the instrument(s) changed over the course of the study	Page 6-7
**Units of study** - Number and relevant characteristics of participants, documents, or events included in the study; level of participation (could be reported in results)	Pages 7-9
**Data processing** - Methods for processing data prior to and during analysis, including transcription, data entry, data management and security, verification of data integrity, data coding, and anonymization/de-identification of excerpts	Page 6-7
**Data analysis** - Process by which inferences, themes, etc., were identified and developed, including the researchers involved in data analysis; usually references a specific paradigm or approach; rationale**	Page 6-7
**Techniques to enhance trustworthiness** - Techniques to enhance trustworthiness and credibility of data analysis (e.g., member checking, audit trail, triangulation); rationale**	Page 7 – double coding.

Results/findings

**Synthesis and interpretation** - Main findings (e.g., interpretations, inferences, and themes); might include development of a theory or model, or integration with prior research or theory	Pages 7 – 14
**Links to empirical data** - Evidence (e.g., quotes, field notes, text excerpts, photographs) to substantiate analytic findings	Pages 9, 11-14

Discussion

**Integration with prior work, implications, transferability, and contribution(s) to the field -** Short summary of main findings; explanation of how findings and conclusions connect to, support, elaborate on, or challenge conclusions of earlier scholarship; discussion of scope of application/generalizability; identification of unique contribution(s) to scholarship in a discipline or field	Pages 14-15
**Limitations** - Trustworthiness and limitations of findings	Page 15

Other

**Conflicts of interest** - Potential sources of influence or perceived influence on study conduct and conclusions; how these were managed	Page 17
**Funding** - Sources of funding and other support; role of funders in data collection, interpretation, and reporting	Page 17

*The authors created the SRQR by searching the literature to identify guidelines, reporting standards, and critical appraisal criteria for qualitative research; reviewing the reference lists of retrieved sources; and contacting experts to gain feedback. The SRQR aims to improve the transparency of all aspects of qualitative research by providing clear standards for reporting qualitative research.

For peer review only

For peer review only - http://bmjopen.bmj.com/site/about/guidelines.xhtml

**The rationale should briefly discuss the justification for choosing that theory, approach, method, or technique rather than other options available, the assumptions and limitations implicit in those choices, and how those choices influence study conclusions and transferability. As appropriate, the rationale for several items might be discussed together.

Reference:

O'Brien BC, Harris IB, Beckman TJ, Reed DA, Cook DA. **Standards for reporting qualitative research: a synthesis of recommendations.** *Academic Medicine*, Vol. 89, No. 9 / Sept 2014 DOI: 10.1097/ACM.0000000000000388

For peer review only

BMJ Open

Acceptability of aspirin for cancer preventive therapy: a survey and qualitative study exploring the views of the UK general population

Journal:	*BMJ Open*
Manuscript ID	bmjopen-2023-078703.R1
Article Type:	Original research
Date Submitted by the Author:	25-Oct-2023
Complete List of Authors:	Lloyd, Kelly; University of Leeds Faculty of Medicine and Health, Leeds Institute of Health Sciences<br>Hall, Louise; University of Leeds Faculty of Medicine and Health, Leeds Institute of Health Sciences<br>Ziegler, Lucy; University of Leeds Faculty of Medicine and Health, Leeds Institute of Health Sciences<br>Foy, Robbie; University of Leeds Faculty of Medicine and Health, Leeds Institute of Health Sciences<br>Green, Sophie M. C.; University of Leeds Faculty of Medicine and Health, Leeds Institute of Health Sciences<br>MacKenzie, Mairead; Independent Cancer Patients' Voice<br>Taylor, David; University College London, Pharmacy<br>Smith, Samuel; University of Leeds Faculty of Medicine and Health, Leeds Institute of Health Sciences
**Primary Subject Heading**:	Public health
Secondary Subject Heading:	Qualitative research, Patient-centred medicine, Oncology, Public health
Keywords:	PREVENTIVE MEDICINE, Behavior, Gastrointestinal tumours < ONCOLOGY, Decision Making

SCHOLARONE™
Manuscripts

I, the Submitting Author has the right to grant and does grant on behalf of all authors of the Work (as defined in the below author licence), an exclusive licence and/or a non-exclusive licence for contributions from authors who are: i) UK Crown employees; ii) where BMJ has agreed a CC-BY licence shall apply, and/or iii) in accordance with the terms applicable for US Federal Government officers or employees acting as part of their official duties; on a worldwide, perpetual, irrevocable, royalty-free basis to BMJ Publishing Group Ltd ("BMJ") its licensees and where the relevant Journal is co-owned by BMJ to the co-owners of the Journal, to publish the Work in this journal and any other BMJ products and to exploit all rights, as set out in our licence.

The Submitting Author accepts and understands that any supply made under these terms is made by BMJ to the Submitting Author unless you are acting as an employee on behalf of your employer or a postgraduate student of an affiliated institution which is paying any applicable article publishing charge ("APC") for Open Access articles. Where the Submitting Author wishes to make the Work available on an Open Access basis (and intends to pay the relevant APC), the terms of reuse of such Open Access shall be governed by a Creative Commons licence – details of these licences and which Creative Commons licence will apply to this Work are set out in our licence referred to above.

Other than as permitted in any relevant BMJ Author's Self Archiving Policies, I confirm this Work has not been accepted for publication elsewhere, is not being considered for publication elsewhere and does not duplicate material already published. I confirm all authors consent to publication of this Work and authorise the granting of this licence.

For peer review only

Title: Acceptability of aspirin for cancer preventive therapy: a survey and qualitative study exploring the views of the UK general population

Authors: Kelly E. Lloyd[1], Louise H. Hall[1], Lucy Ziegler[1], Robbie Foy[1], Sophie M. C. Green[1], Mairead MacKenzie[2], David G. Taylor[3], Samuel G. Smith[1]

Affiliations:

1. Leeds Institute of Health Sciences, University of Leeds, Leeds, UK
2. Independent Cancer Patients' Voice, London, UK
3. School of Pharmacy, University College London, London, UK

Corresponding author: Kelly E. Lloyd, Leeds Institute of Health Science, University of Leeds, Clarendon Way, Leeds, LS2 9NL, UK. Email: k.e.lloyd@leeds.ac.uk. ORCID: 0000-0002-0420-2342.

Key words: Preventive therapy; chemoprevention; cancer prevention; decision-making; aspirin; NSAID

Manuscript word count: 3,806 / 4,000

Abstract word count: 297 / 300

For peer review only - http://bmjopen.bmj.com/site/about/guidelines.xhtml

ABSTRACT

Objectives: Aspirin could be offered for colorectal cancer prevention for the UK general population. To ensure the views of the general population are considered in future guidance, we explored public perceptions of aspirin for preventive therapy.

Design: We conducted an online survey to investigate aspirin use, and awareness of aspirin for cancer prevention among the UK general population. We conducted semi-structured interviews with a sub-sample of survey respondents to explore participants' acceptability towards aspirin for cancer preventive therapy. We analysed the interview data using reflexive thematic analysis, and mapped the themes onto the Theoretical Domains Framework, and the Necessity and Concerns Framework.

Setting: Online survey and remote interviews.

Participants: We recruited 400 UK respondents aged 50-70 through a market research company to the survey. We purposefully sampled, recruited and interviewed 20 survey respondents.

Results: In the survey, 19.0% (76/400) of respondents were aware aspirin can be used to prevent cancer. Among those who had previously taken aspirin, 1.9% (4/216) had taken it for cancer prevention. The interviews generated three themes: 1) Perceived necessity of aspirin; 2) Concerns about side-effects; and 3) Preferred information sources. Participants with a personal or family history of cancer were more likely to perceive aspirin as necessary for cancer prevention. Concerns about taking aspirin at higher doses and its side-effects, such as gastrointestinal bleeding, were common. Many described wanting guidance and advice on aspirin to be communicated from sources perceived as trustworthy, such as healthcare professionals.

Conclusions: Among the general population, those with a personal or family history of cancer may be more receptive towards taking aspirin for preventive therapy. Future policies and campaigns recommending aspirin may be of particular interest to these groups. Multiple considerations about the benefits and risks of aspirin highlight the need to support informed decisions on the medication.

For peer review only - http://bmjopen.bmj.com/site/about/guidelines.xhtml

Strengths and limitations of this study

- The online survey collected unique data on aspirin use and reasons for taking aspirin among the UK general population aged 50 to 70.
- The interviews offered the opportunity to explore the potential barriers and facilitators to taking aspirin for cancer prevention among the general public.
- Self-selection to the survey and interviews may have resulted in recruiting participants with stronger views on aspirin than the wider UK population.

For peer review only

For peer review only - http://bmjopen.bmj.com/site/about/guidelines.xhtml

INTRODUCTION

Colorectal cancer is one of the most common cancers worldwide, with an estimated two million cases and nearly one million deaths from the disease globally in 2020 (1). There is increasing interest in the pharmacological prevention of cancer (2), including aspirin to prevent colorectal cancer (3). A pooled analysis of 423,495 people from two cohort studies found daily aspirin use was associated with a 15% reduced risk of colorectal cancer (hazard ratio: 0.85, 95% CI=0.80-0.89) (4). Furthermore, a meta-analysis of four randomised controlled trials investigating aspirin for vascular disease prevention found aspirin was associated with reduced colon cancer incidence (hazard ratio: 0.76, 95% CI=0.60-0.96) (5). Studies have investigated use of aspirin for preventing other cancers, however the evidence is more limited (6).

Aspirin is often recommended for people at higher risk of developing colorectal cancer. Australian national guidance (7), and the United Kingdom (UK) National Institute for Health and Care Excellence (guidance NG151) recommend aspirin for colorectal cancer prevention among people with Lynch syndrome (8). The guidance does not state a recommended dose, but 150-300mg is commonly used in practice. Australian guidance also recommends considering 100-300mg daily aspirin for those in the general population aged 50 to 70 to prevent colorectal cancer (7). There is no current equivalent national guidance for the public in the UK.

Decisions on whether to use aspirin involve consideration of potential benefits and side-effects, such as an increased risk of gastrointestinal bleeding (9). For individuals at population risk of colorectal cancer, regular aspirin use between 75mg to 325mg appears to have a favourable benefit-harm profile (6), although the risk of side-effects increases substantially after age 70 (6, 10, 11). Current use and acceptability of aspirin for colorectal cancer prevention among the UK public is unknown (12). It is likely though that a proportion of the public have experience taking aspirin (13), such as for pain relief, or cardiovascular disease risk reduction (14). People's prior use of aspirin could support the implementation of the medication for the purpose of colorectal cancer prevention.

Most research examining barriers to using preventive therapy has focused on pharmacological prevention of breast cancer among women at higher risk of the disease. Barriers to use include concerns about side-effects (15-18), and perceptions of the medication as a 'cancer drug' (15). Research has also explored the views of people with Lynch syndrome on aspirin for colorectal cancer prevention (19). Several barriers to taking aspirin were observed, including concerns regarding aspirin's side-effects, and confusion on the recommended dose. It is important to explore the views of the general population on aspirin for cancer prevention, as there are likely to be similar and different barriers to initiating aspirin compared with those at higher risk. Australian cross-sectional

For peer review only - http://bmjopen.bmj.com/site/about/guidelines.xhtml

research has observed moderately high acceptance (>70%) for taking aspirin regularly for colorectal cancer prevention among the general population (20). However, the study did not explore participants' motivators and barriers towards the use of aspirin.

The potential impact of using aspirin for cancer prevention in the wider population will depend on acceptability as well as effectiveness, and an understanding of the barriers to implementation. Public perceptions of aspirin for cancer preventive therapy should be explored to inform both clinical guideline development (21), and support informed decision-making (22). In this study, we aimed to investigate aspirin use and associated knowledge among people from the UK general population. We also aimed to explore the potential facilitators and barriers towards taking aspirin for colorectal cancer prevention.

METHODS

Design

We carried out a survey and qualitative study. We conducted an online survey for two reasons: 1) to collect initial data on participants' prior use of aspirin and their knowledge on taking aspirin for cancer prevention; 2) to recruit people from the UK general population to an interview. Following the survey, we conducted theory-informed, semi-structured, one-to-one interviews with a sub-sample of survey participants to explore their perceptions of aspirin for colorectal cancer prevention. We pre-registered the methods and analysis plan (https://doi.org/10.17605/OSF.IO/3EFG7). We initially preregistered the study to explore the views of people with Lynch syndrome, members of the public, and healthcare professionals on aspirin. We deviated from the protocol by publishing the Lynch syndrome and healthcare professional findings in a separate paper (19). Study reporting was guided by the Standards for Reporting Qualitative Research (SRQR) checklist (23).

Participants and recruitment

We hosted the online survey on Qualtrics, and recruitment was advertised through a market research company (Dynata). We recruited people from the UK public between the ages of 50 to 70, as the benefits of prophylactic aspirin use are estimated to be greater than the risks of side-effects within this age range (6). At the end of the survey, we asked respondents for contact details if they wished to take part in a follow-up interview. All interviewees received £25 from Dynata. One author (KEL) recruited participants until data saturation was considered to have been achieved, following an established method for assessing data saturation (24). Data saturation was used to ensure that the sample size was large enough to provide informative data on the topic, but not so large as to waste resources and participants' time. We used a 10 + 3 stopping criterion to assess for data saturation,

For peer review only - http://bmjopen.bmj.com/site/about/guidelines.xhtml

which is a tested and recommended approach for theory-based interview studies (24). After 10 interviews, we assessed for data saturation and stopped recruitment once three subsequent interviews had been conducted and no new themes were identified (24). For example, recruitment would stop after participant 13, if no new themes were observed from participants 11, 12, and 13.

Survey measures

Aspirin use

We asked participants whether they had ever taken aspirin. Those who answered yes were asked if they took aspirin regularly (i.e. most days or every day) and their reasons for taking aspirin, such as for pain relief, cancer prevention, or cardiovascular disease prevention (Supplementary Materials 1).

Knowledge

We asked participants if they were aware prior to the survey that aspirin can be used to reduce the risk of developing certain cancers. We also asked whether a healthcare provider had previously discussed with them about taking aspirin to prevent colorectal and other cancers.

Characteristics of the sample

We collected data on participant characteristics, including any previous cancer diagnoses, gender, age, ethnicity, and highest educational or professional qualification obtained.

Interview schedule

We developed the interview schedule based on the Theoretical Domains Framework (TDF; version 2) (25), which is a framework derived from multiple behaviour change theories (Supplementary Materials 2). Each of the 14 framework domains describes a factor that could influence individual behaviour when implementing new clinical practices. The TDF domains cover both internal factors, such as a person's knowledge and emotions, and external factors, such as social influences and available resources.

We conducted semi-structured interviews with flexibility to the order of questions, and improvised follow-up questions. At the beginning of all interviews, participants were informed that aspirin is currently only recommended in the UK for people at higher risk of colorectal cancer due to a genetic syndrome, but that there is potential for wider recommended use in the future.

Patient and public involvement

A public representative (MM) reviewed the draft interview schedule to ensure the questions were comprehensible to participants.

For peer review only - http://bmjopen.bmj.com/site/about/guidelines.xhtml

Data collection and analysis

One author (KEL) analysed the survey data in R Studio (R version 4.2.1), with findings presented in proportions and frequencies. One author (KEL), with previous experience in collecting and analysing qualitative data, conducted all interviews by video call or telephone. Interviews took place from July to August 2022. Interviews were audio-recorded, transcribed verbatim, and pseudonymised using initials to replace participants' names.

The interview data was analysed in two stages, which is recommended for the TDF to optimise its use in qualitative research (26), and can identify themes not captured by a theoretical framework (26, 27). The interview transcripts were coded and analysed using reflexive thematic analysis (28, 29). The developed themes were then mapped onto the TDF domains (25). During analysis, we found the Necessity-Concerns Framework to be an additional useful framework for guiding our analytic process (30). The framework specifies that people consider their treatment necessity beliefs against their concerns when making decisions on medication (31, 32), and that these beliefs and concerns on the medication can be general or specific. One author (KEL) coded and analysed all transcripts, and two authors (SGS, SMCG) double coded a proportion of interviews. The findings and potential themes were discussed collaboratively among the three authors, with the aim to explore new perspectives on the data and enhance interpretative depth (33). One author (KEL) mapped the themes onto the domains in the TDF (version 2), which was reviewed by co-authors. All transcripts were managed in NVivo (version 1.6.1) and Microsoft Word.

The survey dataset and analysis scripts generated for the study are publicly available in the Research Data Leeds Repository (34).

RESULTS

Survey findings

Four hundred people participated in the survey (Table 1). The mean age of the sample was 60.8 years (SD=5.7). Most of the sample were men (213; 53.3%), educated below degree level (251; 62.8%), and were white British or Irish (371; 92.8%). Seventy-six (19.0%) were aware prior to the survey that aspirin can be used to reduce the risk of developing certain cancers, and 15 (3.8%) had previously discussed aspirin for this purpose with a healthcare professional.

Most participants (216; 54.0%) had taken aspirin at least once. Among the 216 previous aspirin users, most had taken it for pain relief (150; 69.4%). Fewer had taken aspirin for prevention of cardiovascular disease (61; 28.2%) and cancer (4; 1.9%). One person (0.5%) had taken aspirin as part of a trial. Among the 216 previous aspirin users, 59 (27.3%) reported using aspirin regularly, defined as most days or every day. Most of the 59 regular aspirin users took it for cardiovascular disease

For peer review only - http://bmjopen.bmj.com/site/about/guidelines.xhtml

prevention (45; 76.3%). Other reasons for regularly taking aspirin were pain relief (11/59, 18.6%), and cancer prevention (3/59; 5.1%).

Table 1. Characteristics of the survey respondents, recruited from the UK general population (n=400)

	n **(%)**
**Age**	
50-55	88 (22.0%)
56-60	106 (26.5%)
61-65	103 (25.8%)
66-70	103 (25.8%)
**Gender**	
Women	186 (46.5%)
Men	213 (53.3%)
Another identity	1 (0.3%)
**Ethnicity**	
White British or Irish	371 (92.8%)
White Gypsy or Irish Traveller	1 (0.3%)
Any other White background	12 (3.0%)
Asian or Asian British	9 (2.3%)
Mixed White and Asian	1 (0.3%)
Arab or Arab British	2 (0.5%)
Black/ African/ Caribbean background / mixed background	2 (0.5%)
Any other ethnic background / mixed background	2 (0.5%)
**Education**	
Degree level and above	149 (37.3%)
Below degree level	251 (62.8%)
**Previously diagnosed with cancer**	
Yes	46 (11.5%)
No	354 (88.5%)
**Among those 'Yes' to cancer (*n*=46), which cancer(s)**	
Prostate	11 (23.9%)

For peer review only - http://bmjopen.bmj.com/site/about/guidelines.xhtml

Breast	10 (21.7%)
Skin	6 (13.0%)
Colorectal	5 (10.9%)
Other cancers	14 (30.4%)

Note: Proportions may not compute to 100% due to rounding.

For peer review only

Interview findings

Two hundred and two (50.5%) survey respondents expressed interest in a follow-up interview. We invited participants by email to be interviewed until we judged data saturation was reached. We purposefully sampled participants from different demographic groups (e.g. gender), and a balance of current and never aspirin users. In batches of 5-10 invitations, we invited 53 survey respondents to be interviewed, and 20 (37.7%) responded and were interviewed (Table 2). Interview duration ranged between 15 to 30 minutes. We identified three overarching themes, and interview findings were mapped onto the TDF (Table 3).

Table 2. Characteristics of the general public interview respondents (n = 20)

	***n* (%)**
**Age**	
50-55	3 (15.0%)
56-60	7 (35.0%)
61-65	5 (25.0%)
66-70	5 (25.0%)
**Gender**	
Women	12 (60.0%)
Men	8 (40.0%)
**Ethnicity**	
White British or Irish	17 (85.0%)
Asian or Asian British	3 (15.0%)
**Country in the UK**	
England	18 (90.0%)
Scotland	1 (5.0%)
Northern Ireland	1 (5.0%)
**Previously diagnosed with cancer**	
Yes	6 (30.0%)
No	14 (70.0%)

For peer review only

Table 3. The themes, and corresponding barriers, facilitators, and domains within the Theoretical Domains Framework (TDF; version 2)

**Themes**	**Potential barriers to the use of aspirin for preventive therapy**	**Potential facilitators to the use of aspirin for preventive therapy**	**Main TDF domain(s)**
Perceived necessity of aspirin	Those who perceive themselves at lower risk of cancer because they do not have a personal or family history of the disease.	Those who perceive themselves at higher risk of cancer because they have a personal or family history of the disease.	Beliefs about consequences
Concerns about side-effects	High concerns about using daily aspirin because of the side-effects, such as increased risk of gastrointestinal bleeding.<br>Concerns about taking aspirin daily at doses above 75mg.	Low concerns regarding the side-effects of aspirin because it is a well-known over-the-counter medication for pain relief.	Beliefs about consequences
Preferred information sources	Current lack of a recommendation to support the use of aspirin for colorectal cancer prevention among the UK general public.	Wanting information on aspirin to come from trusted online sources of information, such as the NHS and UK cancer charities (e.g. Cancer Research UK).<br>Wanting information on aspirin to come from a medical professional.	Environmental context and resources

Note. Table adapted from Burgess et al. (35).

For peer review only - http://bmjopen.bmj.com/site/about/guidelines.xhtml

Perceived necessity of aspirin

Did not perceive a need for aspirin

Participant beliefs varied about the perceived necessity of taking aspirin for colorectal cancer prevention. Several people perceived themselves to be at low risk of the disease, typically because they had no family history or personal history of cancer. Often, these participants described how they would only consider using aspirin for cancer prevention if a medical doctor assessed them to be at higher risk, or if they were diagnosed with colorectal cancer.

> *"If someone said to me I was high risk and aspirin was the, was a possible option, I would take it." (J.J., 56-60 years).*

> *"Yeah, well I think probably if I have had bowel cancer and I was in recovery […] then I probably would take [aspirin]." (W.G., 66-70 years).*

Several participants were averse to using daily medication in general, unless deemed highly necessary for their health.

> *"I would only take [medication] if it was really necessary." (A.G., 66-70 years).*

> *"On the whole I don't like taking pills, […]. Unless there was very definite proof that not taking [aspirin] would lead to bowel cancer." (W.G., 66-70 years).*

A few participants perceived other lifestyle changes, such as diet and attending preventive screening, as more effective and necessary than taking medication to prevent cancer.

> *"I think [colorectal cancer] can be broadly governed by diet, fibre, etc." (E.Y., 56-60 years).*

> *"So I'm at the age now where I get a bowel screening, […] So I'm having difficulty seeing what aspirin would add to the mix if I'm kind of okay at the moment." (R.C., 56-60 years).*

Perceived a necessity for aspirin

A number of participants discussed having previously been diagnosed with cancer (e.g., breast, colorectal, prostate), which in turn increased their interest in using aspirin, and their feelings on the importance of cancer prevention.

> *"If I saw the GP yesterday […] 'oh would you like to take aspirin as a preventative for bowel cancer?' I would say yes, particularly now that I've been diagnosed with, with breast cancer." (I.N., 61-65 years).*

For peer review only - http://bmjopen.bmj.com/site/about/guidelines.xhtml

Several participants appeared more inclined to use aspirin because friends or family members had been diagnosed with cancer. All three interviewees who currently used or had previously used aspirin for cancer prevention had started because of a family history of cancer.

> *"A few years ago I heard an article on [a public service radio station] and there was two eminent doctors, and they were talking about the possibility that aspirin prevented not just bowel cancer but other cancers. […] That triggered me to start taking it, my father died of cancer and my sister also died of cancer." (H.B., 66-70 years).*

Concerns about side-effects

Expressed high concerns about aspirin

Many participants expressed concerns about the potential side-effects of daily aspirin use. Several participants expressed feeling more comfortable using a lower dose of daily aspirin (e.g., 75mg), due to the concerns about these side-effects.

> *"Because [150-300mg] seems to be a lot. […], but 75mg, a small dose, I would feel comfortable with that." (S.S., 66-70 years).*

Several participants' concerns regarding aspirin appeared to be related to the perceived harm of taking any long-term medication, and the perception that taking daily medication would mean that they were an 'unhealthy' person.

> *"[From taking medication] you suddenly step from being a healthy person to a potentially unhealthy person because it's a medical intervention" (R.C., 56-60 years).*

Most participants wanted further information on the side-effects of aspirin before they would consider initiating the medication. Participants often wanted to know if the benefits of aspirin for preventive therapy substantially outweighed the side-effects.

> *"I probably would like to know more about what effects it would have on my body, what damage it could do to my heart, or other organs." (K.H., 56-60 years).*

Did not have concerns about aspirin

Despite widespread concerns about aspirin, these views were not universally held among participants. Aspirin was a familiar drug to many, which lowered some concerns about side-effects.

For peer review only - http://bmjopen.bmj.com/site/about/guidelines.xhtml

"Because it's been well known to me that [aspirin] can help prevent heart problems and that's kind of been around for decades […] So I think knowing that and not hearing of anything happening to anybody then that's very reassuring for me." (R.L., 56-60 years).

Some participants expressed low concerns about aspirin because of their own or family members previous experiences using the medication without encountering side-effects.

"No, no I don't [have concerns about the side-effects], because you know whenever I've taken it, […] I don't take them every day but my mother had no side-effects whatsoever." (I.N., 61-65 years).

Preferred information sources

Participants discussed their preferred source of information on the use of aspirin for colorectal cancer prevention. Many participants wanted the information on aspirin to come from trusted online sources, such as the National Health Service (NHS) and UK cancer charities (e.g. Cancer Research UK).

"Cancer Research would probably be, or Macmillan, it would be a trusted website that […] I would look at." (K.H., 56-60 years).

Other important information sources discussed were medical professionals. Several participants wanted the information on aspirin for cancer prevention to come from their GP, while others preferred speaking to a cancer specialist. In most cases, participants felt that they would not initiate aspirin for preventive therapy without a doctor's recommendation.

"Well certainly I think I would first and primarily go to the GP." (A.N., 66-70 years).

"Maybe not a GP but somebody who was specialised in that area, maybe a gastroenterologist […] I wouldn't automatically [take aspirin] because I wouldn't have enough information to warrant what else it could do to me." (K.H., 56-60 years).

Several people expressed positive views towards speaking to a pharmacist about aspirin, often because they had previously had positive experiences consulting their pharmacists on other medical issues. However, not all were comfortable with this approach, and only wanted to speak with a medical doctor about aspirin.

"Well I think my doctor is the trustworthy cause, but also my pharmacy is really good." (A.G., 66-70 years).

For peer review only - http://bmjopen.bmj.com/site/about/guidelines.xhtml

For peer review only

"I've always gone to GP, I've never discussed anything with a pharmacist ever […] I mean they're giving a lot of powers to the pharmacist, but I don't feel comfortable talking to a pharmacist." (L.L., 61-65 years).

Official UK guidance recommending aspirin for cancer prevention for the general public was an important factor to some participants. However, this was only mentioned by a small number of people as a potential barrier to using aspirin.

"I'd be happy if [aspirin] was recommended though, but I'd wait till it was recommended, I wouldn't jump on things too quickly." (F.L., 50-55 years).

DISCUSSION

This study of the UK general population found low reported current use of aspirin for cancer prevention among survey respondents, and several barriers and motivators towards taking aspirin for this purpose across interviewees. In the interviews, those with a personal or family history of cancer were more receptive to taking aspirin whilst those who considered themselves at lower risk of colorectal cancer, or cancer in general, expressed more resistance. Public perceptions towards taking aspirin for cancer prevention should be considered in any future guidance recommending aspirin outside of a Lynch syndrome population.

Most survey respondents had taken aspirin, often for pain relief. People's perceptions of aspirin as a well-known medicine may support its use for prevention, as several participants interviewed held positive views towards aspirin for this reason. However, many had concerns about side-effects, highlighting the need to support informed choices about taking medication. Awareness of aspirin for cancer prevention among survey respondents was low, and only a small percentage took aspirin for prevention. The interview findings suggest family history of cancer was an important motivator to taking aspirin among those at population risk.

We considered our qualitative findings in relation to the 14 domains in the TDF, and identified two main domains (25). These domains were the 'Beliefs about the consequences' of using aspirin, and the potential 'Environmental context and resources' which would aid in implementing aspirin for the public. Participant beliefs about the consequences of taking aspirin, such as the necessity for and side-effects of the medication, were particularly important, and relate to the Necessity-Concerns Framework (30). Several participants expressed specific beliefs and concerns on taking aspirin for cancer prevention, as well as general concerns on taking any medication daily. Previous evidence has found those who report low necessity for a medication and high concerns on the side-effects are less likely to initiate and adhere to a range of different medications (36-39). Similarly, a UK prospective

For peer review only - http://bmjopen.bmj.com/site/about/guidelines.xhtml

study found women at higher risk of breast cancer with low concerns about side-effects were significantly more likely to initiate preventive medication (40).

While the interview findings suggest a relationship between perceived cancer risk and uptake of aspirin, a large population survey is warranted to investigate these potentially motivating factors further. Although minimal previous research has been conducted in this area (12), surveys in the US and Australia have observed mixed findings on a relationship between cancer risk and interest in aspirin for colorectal cancer prevention (20, 41). Interventions aiming to support informed uptake of aspirin for preventive therapy should consider targeting people's beliefs regarding the side-effects and benefits of taking the medication. There is also scope for research to explore the relationship between the Necessity-Concerns Framework and adherence to aspirin.

Guidance recommending aspirin for preventive therapy among the public should consider how the information is communicated, as several participants want the advice to come from a healthcare professional. People who have previously had cancer or a precursor to cancer may be particularly receptive to taking aspirin for preventive therapy. There is trial and observational evidence supporting the use of aspirin among people with colorectal adenomas (42-48), and this may be an appropriate group for policy makers to target if the harm-benefit profile is deemed sufficient. While the evidence for using aspirin for secondary cancer prevention is less developed (49), the ongoing Add-Aspirin trial is investigating the effectiveness of regular aspirin for patients with non-metastatic colorectal, breast, gastro-oesophageal, and prostate cancer (50, 51). Our findings suggest such groups may be particularly receptive to receiving a recommendation to use aspirin for preventive therapy.

Our study had a number of strengths. We utilised two well-established frameworks, the TDF and Necessity-Concerns Framework, to develop a comprehensive analysis of the barriers and motivators towards taking aspirin for colorectal cancer prevention among the general population. A particular strength of the TDF is that the framework can aid in specifying the beliefs and attitudes that are amenable to change (25), and offers an explicit framework for mapping onto behaviour change strategies (e.g., the Behaviour Change Wheel) (52). The online survey also collected unique data on use of daily aspirin and reasons for taking the medication among the UK general population aged 50 to 70.

This study also had limitations. We recruited a moderate sample size of 400 respondents to the survey, therefore the findings should be generalised to the wider UK population with caution. In some cases, weighting approaches can be employed with the aim to weight participants' responses to represent the target population (53). However, we concluded that weighting was inappropriate

For peer review only - http://bmjopen.bmj.com/site/about/guidelines.xhtml

for our survey as several demographic (e.g., ethnicity) categories contained little to no data, which can lead to over or underrepresenting responses for these population groups (54). Self-selection to the survey and interviews may have also resulted in recruiting people with stronger views on the topic than those in the general population (55).

For the interviews, most participants were white, with only three people recruited from an Asian background. Further research is warranted to explore the views of people from different ethnic minority groups on the use of aspirin for cancer preventive therapy. There are likely to be specific barriers among some ethnic minority groups. For example, research has observed South Asian respondents to view cancer as a taboo subject (56, 57), and have discussed the stigma attached to taking long-term medications (58-60), which could reduce uptake of cancer preventive therapy.

CONCLUSION

People with personal or family history of cancer were particularly receptive towards aspirin for colorectal cancer prevention. Future guidance recommending aspirin for cancer prevention may be of particular interest to these groups. Concerns about the side-effects of aspirin were common across the sample, highlighting the need to support informed decisions on the medication. Guidance and advice recommending aspirin should be communicated from sources perceived as trustworthy by the public, such as healthcare professionals.

For peer review only - http://bmjopen.bmj.com/site/about/guidelines.xhtml

DECLARATIONS

Acknowledgements: We thank all the participants who took part in the study. We also acknowledge the AsCaP senior executive board and committee members.

Ethical approval: Ethical approval was awarded by the University of Leeds School of Medicine Research Ethics Committee (MREC 19-091). Verbal informed consent was obtained from all individual participants included in this study.

Competing interests: Robbie Foy is a member of the NICE Implementation Strategy Group. All other authors have no competing interests to declare that are relevant to the content of this article.

Funding: This work is fully funded by the Aspirin for Cancer Prevention AsCaP Group CRUK Grant Code: **A24991,** Senior Executive Board Prof. J Burn, Prof. A.T Chan, Prof. J Cuzick, Dr. B Nedjai, Prof. Ruth Langley. During the study, KEL was supported by an Economic and Social Research Council studentship [grant number ES/P000745/1]. KEL also acknowledges funding support from an ESRC Postdoctoral Fellowship [ES/Y00759X/1]. This report is independent research supported by the National Institute for Health Research NIHR Advanced Fellowship, Professor Samuel Smith, [grant number NIHR300588]. SGS also acknowledges funding support from a Yorkshire Cancer Research University Academic Fellowship. The funders had no role in study design, data collection and analysis, decision to publish, or preparation of the manuscript.

Data sharing statement: The survey data is available in a public, open access repository. The survey dataset and analysis scripts are publicly available in the Research Data Leeds Repository: https://doi.org/10.5518/1379. The qualitative data is available upon reasonable request. The methods and analysis plan were pre-registered: https://doi.org/10.17605/OSF.IO/3EFG7.

Author contributions: Conceptualisation: K.E.L, S.G.S, R.F, L.H.H, and L.Z. Methodology: K.E.L, S.G.S, R.F, L.H.H, L.Z, D.G.T, and M.M. Supervision: S.G.S, R.F, L.H.H, and L.Z. Funding acquisition: S.G.S, D.G.T, and M.M. Investigation: K.E.L. Formal analysis: K.E.L, S.M.C.G, and S.G.S. Writing – original draft: K.E.L. Writing – review and editing: all authors.

Aspirin for Cancer Prevention AsCaP Steering Committee Members:

Professor Jack Cuzick, Queen Mary University of London. Chair

Professor Frances Balkwill, Queen Mary University of London

Professor Tim Bishop, University of Leeds

Professor Sir John Burn, Newcastle University

For peer review only - http://bmjopen.bmj.com/site/about/guidelines.xhtml

Professor Andrew T. Chan, Harvard School of Medicine

Dr Colin Crooks, University of Nottingham

Professor Chris Hawkey, University of Nottingham

Professor Ruth Langley, University College London

Ms Mairead McKenzie, Independent Cancer Patients' Voice

Dr Belinda Nedjai, Queen Mary University of London

Professor Paola Patrignani, Università "G. d'Annunzio" di Chieti-Pescara

Professor Carlo Patrono, Catholic University of the Sacred Heart, Rome

Dr Bianca Rocca, Catholic University of the Sacred Heart, Rome

Professor Samuel Smith, University of Leeds

Dr Laura Greaves, Newcastle University

For peer review only

For peer review only - http://bmjopen.bmj.com/site/about/guidelines.xhtml

REFERENCES

1. Xi Y, Xu P. Global colorectal cancer burden in 2020 and projections to 2040. Transl Oncol. 2021;14 (10):101174.

2. Benetou V, Lagiou A, Lagiou P. Chemoprevention of cancer: current evidence and future prospects. F1000Res. 2015;4 (F1000 Faculty Rev):916.

3. Katona BW, Weiss JM. Chemoprevention of Colorectal Cancer. Gastroenterology. 2020;158 (2):368-88.

4. Hurwitz LM, Michels KA, Cook MB, Pfeiffer RM, Trabert B. Associations between daily aspirin use and cancer risk across strata of major cancer risk factors in two large U.S. cohorts. Cancer Causes Control. 2021;32 (1):57-65.

5. Rothwell PM, Wilson M, Elwin CE, Norrving B, Algra A, Warlow CP, et al. Long-term effect of aspirin on colorectal cancer incidence and mortality: 20-year follow-up of five randomised trials. Lancet. 2010;376 (9754):1741-50.

6. Cuzick J, Thorat MA, Bosetti C, Brown PH, Burn J, Cook NR, et al. Estimates of benefits and harms of prophylactic use of aspirin in the general population. Ann oncol. 2015;26 (1):47-57.

7. Cancer Council Australia. Chemopreventive candidate agents. [Online]. 2019 [Accessed August 2022]. Available from: https://wiki.cancer.org.au/australia/Clinical_question:Aspirin_for_prevention_of_colorectal_cancer.

8. National Institute for Health and Clinical Excellence (NICE). Colorectal cancer [NG151]. [Online]. 2020. [Accessed August 2022]. Available from: https://www.nice.org.uk/guidance/NG151.

9. Sørensen HT, Mellemkjær L, Blot WJ, Nielsen GL, Steffensen FH, McLaughlin JK, et al. Risk of upper gastrointestinal bleeding associated with use of low-dose aspirin. Am J Gastroenterol. 2000;95 (9):2218-24.

10. McNeil JJ, Wolfe R, Woods RL, Tonkin AM, Donnan GA, Nelson MR, et al. Effect of Aspirin on Cardiovascular Events and Bleeding in the Healthy Elderly. N Engl J Med. 2018;379 (16):1509-18.

11. McNeil JJ, Gibbs P, Orchard SG, Lockery JE, Bernstein WB, Cao Y, et al. Effect of Aspirin on Cancer Incidence and Mortality in Older Adults. J Natl Cancer Inst. 2021;113 (3):258-65.

12. Lloyd KE, Hall LH, King N, Thorneloe RJ, Rodriguez-Lopez R, Ziegler L, et al. Aspirin use for cancer prevention: A systematic review of public, patient and healthcare provider attitudes and adherence behaviours. Prev Med. 2021;154:106872.

For peer review only - http://bmjopen.bmj.com/site/about/guidelines.xhtml

13. Trinder P, Rajaratnam G, Lewis M, Croft P. Prophylactic aspirin use in the adult general population. J Public Health Med. 2003;25 (4):377-80.

14. Campbell CL, Smyth S, Montalescot G, Steinhubl SR. Aspirin dose for the prevention of cardiovascular disease: a systematic review. JAMA. 2007;297 (18):2018-24.

15. Donnelly LS, Evans DG, Wiseman J, Fox J, Greenhalgh R, Affen J, et al. Uptake of tamoxifen in consecutive premenopausal women under surveillance in a high-risk breast cancer clinic. Br J Cancer. 2014;110 (7):1681-7.

16. Heisey R, Pimlott N, Clemons M, Cummings S, Drummond N. Women's views on chemoprevention of breast cancer: qualitative study. Can Fam Physician. 2006;52 (5):624-5.

17. Altschuler A, Somkin CP. Women's Decision Making About Whether or Not to Use Breast Cancer Chemoprevention. Women Health. 2005;41 (2):81-95.

18. Hackett J, Thorneloe R, Side L, Wolf M, Horne R, Cuzick J, et al. Uptake of breast cancer preventive therapy in the UK: results from a multicentre prospective survey and qualitative interviews. Breast Cancer Res Treat. 2018;170 (3):633-40.

19. Lloyd KE, Foy R, Hall LH, Ziegler L, Green SMC, Haider ZF, et al. Barriers and facilitators to using aspirin for preventive therapy: a qualitative study exploring the views and experiences of people with Lynch syndrome and healthcare providers. Hered Cancer Clin Pract. 2022;20 (1):30.

20. Nguyen P, McIntosh J, Bickerstaffe A, Maddumarachchi S, Cummings K-L, Emery JD. Benefits and harms of aspirin to reduce colorectal cancer risk: a cross-sectional study of methods to communicate risk in primary care. Br J Gen Pract. 2019;69(689):e843.

21. Boivin A, Currie K, Fervers B, Gracia J, James M, Marshall C, et al. Patient and public involvement in clinical guidelines: international experiences and future perspectives. Qual Saf Health Care. 2010;19 (5):e22.

22. Stiggelbout AM, Weijden TVd, Wit MPTD, Frosch D, Légaré F, Montori VM, et al. Shared decision making: really putting patients at the centre of healthcare. BMJ. 2012;344:e256.

23. O'Brien BC, Harris IB, Beckman TJ, Reed DA, Cook DA. Standards for Reporting Qualitative Research: a synthesis of recommendations. Acad Med. 2014;89(9).

For peer review only - http://bmjopen.bmj.com/site/about/guidelines.xhtml

24. Francis JJ, Johnston M, Robertson C, Glidewell L, Entwistle V, Eccles MP, et al. What is an adequate sample size? Operationalising data saturation for theory-based interview studies. Psychol Health. 2010;25 (10):1229-45.

25. Atkins L, Francis J, Islam R, O'Connor D, Patey A, Ivers N, et al. A guide to using the Theoretical Domains Framework of behaviour change to investigate implementation problems. Implement Sci. 2017;12 (1):77.

26. McGowan LJ, Powell R, French DP. How can use of the Theoretical Domains Framework be optimized in qualitative research? A rapid systematic review. Br J Health Psychol. 2020;25 (3):677-94.

27. MacFarlane A, O'Reilly-de Brún M. Using a theory-driven conceptual framework in qualitative health research. Qual Health Res. 2012;22 (5):607-18.

28. Braun V, Clarke V. One size fits all? What counts as quality practice in (reflexive) thematic analysis? Qual Res Psychol. 2021;18 (3):328-52.

29. Braun V, Clarke V. Using thematic analysis in psychology. Qual Res Psychol. 2006;3 (2):77-101.

30. Horne R, Weinman J, Hankins M. The beliefs about medicines questionnaire: The development and evaluation of a new method for assessing the cognitive representation of medication. Psychol Health. 1999;14 (1):1-24.

31. Horne R, Chapman SCE, Parham R, Freemantle N, Forbes A, Cooper V. Understanding patients' adherence-related beliefs about medicines prescribed for long-term conditions: a meta-analytic review of the necessity-concerns framework. PLOS ONE. 2013;8 (12):e80633.

32. Phillips LA, Diefenbach MA, Kronish IM, Negron RM, Horowitz CR. The Necessity-Concerns Framework: a multidimensional theory benefits from multidimensional analysis. Ann Behav Med. 2014;48 (1):7-16.

33. Braun V, Clarke V. Conceptual and design thinking for thematic analysis. Qual Psychol. 2022 Feb;9(1):3.

[Dataset] 34. Lloyd KE, Hall LH, Ziegler L, Foy R, Green SMC, MacKenzie M, et al. Data from: Short survey on aspirin use for cancer prevention among the UK general public. Research Data Leeds Repsoitory. July 14, 2023. https://doi.org/10.5518/1379

For peer review only - http://bmjopen.bmj.com/site/about/guidelines.xhtml

35. Burgess C, Wright AJ, Forster AS, Dodhia H, Miller J, Fuller F, et al. Influences on individuals' decisions to take up the offer of a health check: a qualitative study. Health Expect. 2015;18 (6):2437-48.

36. Clifford S, Barber N, Horne R. Understanding different beliefs held by adherers, unintentional nonadherers, and intentional nonadherers: application of the Necessity–Concerns Framework. J Psychosom Res. 2008;64 (1):41-6.

37. Horne R, Cooper V, Gellaitry G, Date HL, Fisher M. Patients' perceptions of highly active antiretroviral therapy in relation to treatment uptake and adherence: the utility of the Necessity-Concerns Framework. JAIDS-J ACQ IMM DEF. 2007;45(3).

38. Horne R, Parham R, Driscoll R, Robinson A. Patients' attitudes to medicines and adherence to maintenance treatment in inflammatory bowel disease. Inflamm Bowel Dis. 2009;15 (6):837-44.

39. Clarkesmith DE, Pattison HM, Lip GYH, Lane DA. Educational intervention improves anticoagulation control in atrial fibrillation patients: the TREAT randomised trial. PLOS ONE. 2013;8 (9):e74037.

40. Thorneloe RJ, Horne R, Side L, Wolf MS, Smith SG, Adamson V, et al. Beliefs about medication and uptake of preventive therapy in women at increased risk of breast cancer: results from a multicenter prospective study. Clin Breast Cancer. 2019;19 (1):e116-e26.

41. Jensen JD, Holton AE, Krakow M, Weaver J, Donovan E, Tavtigian S. Colorectal cancer prevention and intentions to use low-dose aspirin: A survey of 1000 U.S. adults aged 40–65. Cancer Epidemiol. 2016;41:99-105.

42. Logan RF, Grainge MJ, Shepherd VC, Armitage NC, Muir KR. Aspirin and folic acid for the prevention of recurrent colorectal adenomas. Gastroenterology. 2008;134 (1):29-38.

43. Sandler RS, Halabi S, Baron JA, Budinger S, Paskett E, Keresztes R, et al. A randomized trial of aspirin to prevent colorectal adenomas in patients with previous colorectal cancer. N Engl J Med. 2003;348 (10):883-90.

44. Baron JA, Cole BF, Sandler RS, Haile RW, Ahnen D, Bresalier R, et al. A randomized trial of aspirin to prevent colorectal adenomas. N Engl J Med. 2003;348 (10):891-9.

45. Benamouzig R, Deyra J, Martin A, Girard B, Jullian E, Piednoir B, et al. Daily soluble aspirin and prevention of colorectal adenoma recurrence: one-year results of the apacc trial. Gastroenterology. 2003;125 (2):328-36.

For peer review only

For peer review only - http://bmjopen.bmj.com/site/about/guidelines.xhtml

46. Cole BF, Logan RF, Halabi S, Benamouzig R, Sandler RS, Grainge MJ, et al. Aspirin for the chemoprevention of colorectal adenomas: meta-analysis of the randomized trials. J Natl Cancer Inst. 2009;101 (4):256-66.

47. Grau MV, Sandler RS, McKeown-Eyssen G, Bresalier RS, Haile RW, Barry EL, et al. Nonsteroidal anti-inflammatory drug use after 3 years of aspirin use and colorectal adenoma risk: observational follow-up of a randomized study. J Natl Cancer Inst. 2009;101 (4):267-76.

48. Chudy-Onwugaje K, Huang W-Y, Su LJ, Purdue MP, Johnson CC, Wang L, et al. Aspirin, ibuprofen, and reduced risk of advanced colorectal adenoma incidence and recurrence and colorectal cancer in the PLCO Cancer Screening Trial. Cancer. 2021;127 (17):3145-55.

49. Langley RE, Burdett S, Tierney JF, Cafferty F, Parmar MKB, Venning G. Aspirin and cancer: has aspirin been overlooked as an adjuvant therapy? Br J Cancer. 2011;105 (8):1107-13.

50. Coyle C, Cafferty FH, Rowley S, MacKenzie M, Berkman L, Gupta S, et al. ADD-ASPIRIN: A phase III, double-blind, placebo controlled, randomised trial assessing the effects of aspirin on disease recurrence and survival after primary therapy in common non-metastatic solid tumours. Contemp clin trials. 2016;51:56-64.

51. Joharatnam-Hogan N, Cafferty F, Hubner R, Swinson D, Sothi S, Gupta K, et al. Aspirin as an adjuvant treatment for cancer: feasibility results from the Add-Aspirin randomised trial. Lancet Gastroenterol Hepatol. 2019;4 (11):854-62.

52. Michie S, Atkins L, West R. *The behaviour change wheel: A guide to designing interventions.* London: Silverback Publishing. 2014.

53. Kalton G, Flores-Cervantes I. Weighting methods. J Off Stat. 2003;19 (2):81.

54. Vaske JJ, Jacobs MH, Sijtsma MT, Beaman J. Can weighting compensate for sampling issues in internet surveys? Hum Dimens Wildl. 2011;16 (3):200-15.

55. Comley P, Beaumont J. Online market research: Methods, benefits and issues — Part 1. J Direct Data Digit Mark Pract. 2011;12(4):315-27.

56. Randhawa G, Owens A. The meanings of cancer and perceptions of cancer services among South Asians in Luton, UK. Br J Cancer. 2004;91 (1):62-8.

57. Karbani G, Lim JN, Hewison J, Atkin K, Horgan K, Lansdown M, et al. Culture, attitude and knowledge about breast cancer and preventive measures: a qualitative study of South Asian breast cancer patients in the UK. Asian Pac J Cancer Prev. 2011;12 (6):1619-26.

For peer review only - http://bmjopen.bmj.com/site/about/guidelines.xhtml

58. Kumar K, Greenfield S, Raza K, Gill P, Stack R. Understanding adherence-related beliefs about medicine amongst patients of South Asian origin with diabetes and cardiovascular disease patients: a qualitative synthesis. BMC Endocr Disord. 2016;16 (1):24.

59. Ens TA, Seneviratne CC, Jones C, King-Shier KM. Factors influencing medication adherence in South Asian people with cardiac disorders: an ethnographic study. Int J Nurs Stud. 2014;51 (11):1472-81.

60. Kamath DY, Bhuvana K, Salazar LJ, Varghese K, Kamath A, Idiculla J, et al. A qualitative, grounded theory exploration of the determinants of self-care behavior among Indian patients with a lived experience of chronic heart failure. PLOS ONE. 2021;16 (1):e0245659.

For peer review only

Supplementary Materials 1: Short survey on aspirin use for cancer prevention among the general public

1. Do you ever take aspirin?
    a. Yes
    b. No

2. Do you take aspirin regularly (i.e. most days or every day)?
    a. Yes
    b. No
    c. N/A – do not take aspirin

3. What is the main reason you take aspirin?
    a. Pain relief
    b. Prevention of cardiovascular disease (i.e. heart and circulatory disease)
    c. Prevention of cancer
    d. Other reason, please specify ______________
    e. N/A – do not take aspirin

4. Prior to completing this survey, were you aware that aspirin could reduce the risk of developing certain types of cancers?
    a. Yes
    b. No

5. Has your doctor or any other healthcare professional talked to you about how aspirin may lower your risk of developing bowel cancer or any other cancers?
    a. Yes
    b. No

6. Have you previously been diagnosed with cancer?
    a. Yes, please specify which cancer(s): _______________
    b. No

7. How would you describe your gender?
    a. Male
    b. Female
    c. Non-binary
    d. Different identity
    e. Prefer not to say

8. What is your age?

    ____

9. How would you describe your ethnicity?
    a. White - English/ Welsh/ Scottish/ Northern Irish/ British
    b. White - Irish

For peer review only

c. White - Gypsy or Irish Traveller
d. Any other White background
e. Mixed - White and Black Caribbean
f. Mixed - White and Black African
g. Mixed - White and Asian
h. Any other mixed/ multiple ethnic background
i. Asian or Asian British - Indian
j. Asian or Asian British - Pakistani
k. Asian or Asian British - Bangladeshi
l. Asian or Asian British - Chinese
m. Any other Asian background
n. Black or Black British - African
o. Black or Black British - Caribbean
p. Any other Black/ African/ Caribbean background
q. Arab or Arab British
r. Any other ethnic group

10. What is the highest level of educational or professional qualification you have obtained?
    a. GCSE/ O-level/ CSE
    b. Vocational qualifications (e.g. NVQ1+2)
    c. A-Level or equivalent (e.g. NVQ3)
    d. Bachelor's degree or equivalent (e.g. NVQ4)
    e. Masters/ PhD or equivalent
    f. Other qualifications
    g. No formal qualifications

Thank you for taking part in the survey study. If you are interested in taking part further, we are looking for people to take part in a follow-up interview study to explore their thoughts on the use of aspirin for bowel cancer prevention. The interviews will take between 30 to 60 minutes to complete and will be take place either over the telephone or video call, depending upon your preference. You will receive £25 for taking part in the follow-up interview study. You do not need to use aspirin or know anything about this topic to take part.

11. Are you interested in taking part in the follow-up interview study?
    a. Yes, please provide your contact details (e.g. email, phone number) and we will contact you further about the study: ___________________
    b. No

For peer review only

Supplementary Materials 2: Interview schedule for general public interviews

Thank you for agreeing to take part. Just before we start the interview, I'd like to quickly go over some key points about your rights as a participant in this study. It is completely fine if at any point you would like to stop the interview, or if you do not want to answer a question, please just let me know. You can also withdraw from the study at any point and you do not have to provide a reason for why. Just to remind you the interview will be recorded, but anything you say to me today will be kept confidential, and if you are quoted in any of our reports your name will not be used and instead you will be given a pseudonym, which is a fake name. I will also anonymise anything identifiable, like places, you mention as well. After the interview, if you have any further questions about anything we discussed today, please feel free to contact me. I can also provide a debrief sheet at the end with website links with further information on this topic in case you are interested in reading more. Are you happy to continue?

General public interview questions

As you read on the information sheet, the study is aiming to understand what people think about using aspirin for cancer prevention, including the reasons why people may or may not be willing to use it. Aspirin has previously been found to effectively reduce people's risk of bowel cancer in a healthy population sample. At the moment, aspirin is only officially recommended by the NHS for cancer prevention in people who have a genetic condition that makes them higher risk of developing bowel cancer. There is potential in the future though for this medication to be offered more widely to the general public for the purpose of bowel cancer prevention.

While there is the potential benefit of having a reduced likelihood of developing bowel cancer in the future, there are also side-effects to regular aspirin use. The main side-effect is an increased likelihood of experiencing internal bleeding.

I will now move onto the interview questions, which will explore your thoughts on using aspirin regularly for cancer prevention. Just a reminder before we start, you do not need to know anything about the use of aspirin for cancer prevention to answer these questions. We are just interested in hearing your initial thoughts.

For peer review only

**Domains**	**Interview questions**
Knowledge<br><br>Enviromental context and resources<br><br>Intentions<br><br>Social influences	What are your initial thoughts on taking aspirin daily for bowel cancer prevention?<br><br>Had you heard of the use of aspirin for cancer prevention before this interview?<br><br>If yes, where and how did you hear about this?<br><br>If no, how would you find such information? Prompts: from what sources? Internet? A healthcare professional?<br><br>Have you ever been encouraged or discouraged by someone to take aspirin regularly for cancer prevention? Prompt: Why was this?
Optimism<br><br>Beliefs about consequences<br><br>Emotions<br><br>Enviromental context and resources<br><br>Skills<br><br>Beliefs about capabilities<br><br>Reinforcement	In your opinion, do you think using aspirin would be a good way to reduce your risk of developing bowel cancer in the future? Prompt: Why do you think this?<br><br>Do you have any concerns about taking aspirin regularly? Prompt: How do you feel about the side-effects?<br><br>What kind of information do you think you would need to help you make a decision on whether to take aspirin regularly for cancer prevention?<br><br>Where would you go to get this information? Prompts: What would your first step be to obtain this information? Internet? Healthcare professionals?<br><br>*[If they do take aspirin for cancer prevention]* Have you previously encountered any problems with trying to get further information on the use of aspirin for cancer prevention? If yes, please describe these problems.<br><br>*[If yes to above question]* Is there anything about this experience that makes you more or less likely to try and take aspirin regularly in the future?
Beliefs about consequences	I mentioned before one of potential side-effects of regular aspirin use can be internal bleeding. There are several risk factors that make a person more likely to experience internal bleeding from regular aspirin use. These include:<br><br>- Active or previous stomach ulcers<br>- Bleeding disorders

For peer review only

	- Being over the age of 65<br>- Uncontrolled hypertension<br>- Previous history of stroke<br>- Abnormal liver or renal function<br>- Experience indigestion on aspirin<br><br>If you had any of these risk factors, your doctor may offer you an additional medication to reduce your risk of internal bleeding.<br><br>What are your thoughts on taking regular aspirin alongside another medication to reduce your risk of bowel cancer?
Goals<br><br>Memory, attention and decision processes	How much of a priority is taking aspirin for cancer prevention to you? Prompts: how high or low a priority is it?<br><br>Are there any other higher priorities? Prompt: Prevention priorities? What are they?

Brief quantitative questions

Thank you so much for your answers so far. Just before we end the interview, there are several brief demographic questions that I would just like to go through with you. If you do not want to answer one of these questions, please just let me know and we can skip it.

1) What is your age?
2) Please describe your gender? Male; Female; Non-binary; Another identity; Prefer not to say.
3) How would you describe your ethnicity? Examples include White British, Indian, Mixed – White and Black Caribbean
4) What country in the UK do you live?
5) Have you previously been diagnosed with cancer?

Debrief

Thank you for all your help with answering my questions. We really appreciate your time and hope that it will be useful in the future when we are trying to support people when making a decision about whether to use aspirin for cancer prevention. Before we end, do you have any questions you would like to ask me?

For peer review only

If you would like more information on the use of aspirin for cancer prevention, I can email you an information sheet with links to website with further information on this topic. If you would like to ask me further questions on the study, please do not hesitate to contact me.

For peer review only

Standards for Reporting Qualitative Research (SRQR)*

http://www.equator-network.org/reporting-guidelines/srqr/

Title and abstract

	Page/line no(s).
**Title** - Concise description of the nature and topic of the study Identifying the study as qualitative or indicating the approach (e.g., ethnography, grounded theory) or data collection methods (e.g., interview, focus group) is recommended	Page 1
**Abstract** - Summary of key elements of the study using the abstract format of the intended publication; typically includes background, purpose, methods, results, and conclusions	Page 2

Introduction

**Problem formulation** - Description and significance of the problem/phenomenon studied; review of relevant theory and empirical work; problem statement	Page 4-5
**Purpose or research question** - Purpose of the study and specific objectives or questions	Page 5

Methods

**Qualitative approach and research paradigm** - Qualitative approach (e.g., ethnography, grounded theory, case study, phenomenology, narrative research) and guiding theory if appropriate; identifying the research paradigm (e.g., postpositivist, constructivist/ interpretivist) is also recommended; rationale**	Page 6-7<br>Theories: Theoretical Domains Framework, and Necessity-Concerns Framework
**Researcher characteristics and reflexivity** - Researchers' characteristics that may influence the research, including personal attributes, qualifications/experience, relationship with participants, assumptions, and/or presuppositions; potential or actual interaction between researchers' characteristics and the research questions, approach, methods, results, and/or transferability	Page 6 – interviewer's previous qualitative experience discussed
**Context** - Setting/site and salient contextual factors; rationale**	Page 5 – online survey<br>Page 6 – telephone or video interview
**Sampling strategy** - How and why research participants, documents, or events were selected; criteria for deciding when no further sampling was necessary (e.g., sampling saturation); rationale**	Page 5 – eligibility and rationale.<br>Page 5 and page 9 – data saturation.
**Ethical issues pertaining to human subjects** - Documentation of approval by an appropriate ethics review board and participant consent, or explanation for lack thereof; other confidentiality and data security issues	Declarations section
**Data collection methods** - Types of data collected; details of data collection procedures including (as appropriate) start and stop dates of data collection and analysis, iterative process, triangulation of sources/methods, and modification of procedures in response to evolving study findings; rationale**	Pages 5 to 7.<br>Interview schedule – supplementary materials.

For peer review only - http://bmjopen.bmj.com/site/about/guidelines.xhtml

For peer review only

**Data collection instruments and technologies** - Description of instruments (e.g., interview guides, questionnaires) and devices (e.g., audio recorders) used for data collection; if/how the instrument(s) changed over the course of the study	Page 6-7
**Units of study** - Number and relevant characteristics of participants, documents, or events included in the study; level of participation (could be reported in results)	Pages 7-9
**Data processing** - Methods for processing data prior to and during analysis, including transcription, data entry, data management and security, verification of data integrity, data coding, and anonymization/de-identification of excerpts	Page 6-7
**Data analysis** - Process by which inferences, themes, etc., were identified and developed, including the researchers involved in data analysis; usually references a specific paradigm or approach; rationale**	Page 6-7
**Techniques to enhance trustworthiness** - Techniques to enhance trustworthiness and credibility of data analysis (e.g., member checking, audit trail, triangulation); rationale**	Page 7 – double coding.

Results/findings

**Synthesis and interpretation** - Main findings (e.g., interpretations, inferences, and themes); might include development of a theory or model, or integration with prior research or theory	Pages 7 – 14
**Links to empirical data** - Evidence (e.g., quotes, field notes, text excerpts, photographs) to substantiate analytic findings	Pages 9, 11-14

Discussion

**Integration with prior work, implications, transferability, and contribution(s) to the field -** Short summary of main findings; explanation of how findings and conclusions connect to, support, elaborate on, or challenge conclusions of earlier scholarship; discussion of scope of application/generalizability; identification of unique contribution(s) to scholarship in a discipline or field	Pages 14-15
**Limitations** - Trustworthiness and limitations of findings	Page 15

Other

**Conflicts of interest** - Potential sources of influence or perceived influence on study conduct and conclusions; how these were managed	Page 17
**Funding** - Sources of funding and other support; role of funders in data collection, interpretation, and reporting	Page 17

*The authors created the SRQR by searching the literature to identify guidelines, reporting standards, and critical appraisal criteria for qualitative research; reviewing the reference lists of retrieved sources; and contacting experts to gain feedback. The SRQR aims to improve the transparency of all aspects of qualitative research by providing clear standards for reporting qualitative research.

For peer review only

For peer review only - http://bmjopen.bmj.com/site/about/guidelines.xhtml

**The rationale should briefly discuss the justification for choosing that theory, approach, method, or technique rather than other options available, the assumptions and limitations implicit in those choices, and how those choices influence study conclusions and transferability. As appropriate, the rationale for several items might be discussed together.

Reference:
O'Brien BC, Harris IB, Beckman TJ, Reed DA, Cook DA. **Standards for reporting qualitative research: a synthesis of recommendations.** *Academic Medicine*, Vol. 89, No. 9 / Sept 2014 DOI: 10.1097/ACM.0000000000000388

For peer review only
